# Tutorial Review of Bio-Inspired Approaches to Robotic Manipulation for Space Debris Salvage

**DOI:** 10.3390/biomimetics5020019

**Published:** 2020-05-12

**Authors:** Alex Ellery

**Affiliations:** Department of Mechanical & Aerospace Engineering, Carleton University, 1125 Colonel by Drive, Ottawa, ON K1S 5B6, Canada; AlexEllery@CUNET.CARLETON.CA

**Keywords:** space debris mitigation, on-orbit servicing, space salvage, predictive forward models, cerebellum, preflex, viscoelastic muscle

## Abstract

We present a comprehensive tutorial review that explores the application of bio-inspired approaches to robot control systems for grappling and manipulating a wide range of space debris targets. Current robot manipulator control systems exploit limited techniques which can be supplemented by additional bio-inspired methods to provide a robust suite of robot manipulation technologies. In doing so, we review bio-inspired control methods because this will be the key to enabling such capabilities. In particular, force feedback control may be supplemented with predictive forward models and software emulation of viscoelastic preflexive joint behaviour. This models human manipulation capabilities as implemented by the cerebellum and muscles/joints respectively. In effect, we are proposing a three-level control strategy based on biomimetic forward models for predictive estimation, traditional feedback control and biomimetic muscle-like preflexes. We place emphasis on bio-inspired forward modelling suggesting that all roads lead to this solution for robust and adaptive manipulator control. This promises robust and adaptive manipulation for complex tasks in salvaging space debris.

## 1. Introduction

The problem of space debris imposes a severe and present danger to current and future space operations. Furthermore, the space debris problem is becoming more acute with increasing numbers of satellites launched—in 2017, 400 satellites were launched, over four times the annual average from 2000–2010. Recently, SpaceX launched 60 Starlink satellites into 550 km altitude, the first of a megaconstellation of 7518 satellites into non-geosychronous orbits to bring broadband internet services globally to ensure full coverage for the 3.5 B people currently without. Telesat intends to launch a 512-satellite constellation while OneWeb intends to launch a 900-satellite constellation. Plans envisage expanding satellite constellations to 40,000. Multitudes of satellites at lower altitude reduce signal propagation time compared with geosynchronous orbit. Yet, thus far, only 9000 satellites have been launched in the history of spaceflight. Although 550 km altitude is sufficiently low for orbital decay due to atmospheric braking, these broadband internet constellations vastly increase the prospects for collision and the rapid accumulation of debris. The space debris problem has become critical and requires serious intervention to address it. It has been suggested that if 5–10 of the largest defunct satellites were disposed of annually in LEO (low Earth orbit) where space debris is most concentrated, this would prevent the Kessler syndrome from occurring [1]—the Kessler limit occurs when the rate of fragmentation of debris runs away and becomes uncontrollable [2]. It is believed that we may be approaching the Kessler limit in polar low-Earth orbit. There is, although most debris burn up in the atmosphere on re-entry, the finite prospect of being struck on Earth by surviving debris from space. In 1997, a woman in Oklahoma was struck without injury by a piece of launch shroud but most re-entering debris falls into the oceans. Nevertheless, the vastly expanding satellite population will increase this risk of human exposure. Unfortunately, the UN Liability Convention (1972) in conjunction with the UN Registration Convention (1976) do not appear to be effective—part of the problem lies in proving fault by establishing demonstrable causation by specific debris. The Chinese and Russian responsibility for the criticality of the space debris environment suggests that recent ISO standards recommending satellite disposal at the end of life are likely to be useless, suggesting technological solutions. 

Surrey Space Centre’s RemoveDEBRIS technology demonstrator mission demonstrated four techniques on two companion inflatable cubesats—weighted net capture, laser scanning to extract shape, harpooning a target plate and unfurling a drag sail to demonstrate fuel-less disposal by de-orbiting. It successfully demonstrated the two capture methods. There are several concerns about these approaches, not least being their propensity for generating secondary debris. The most favoured methods of space debris acquisition—harpooning and netting—ultimately involve disposal, introducing the problem of re-entry survivability and controllability, particularly for a large defunct spacecraft like Envisat. It is possible that a controlled re-entry can direct debris into the Pacific Ocean between Chile and New Zealand where there are few aircraft flights and only rarely used shipping lanes. This requires a steep and controlled descent which consumes considerable fuel. Most defunct spacecraft will require retro-fitting with a de-orbit device—if passive (such as a drag-sail), the re-entry is only partially controlled. The European Space Agency decided to redesign its e.Deorbit mission, originally for removing Envisat, to accommodate a robotic arm for its multiple utility for on-orbit servicing. One of the criticisms of e.Deorbit and other debris-specific solutions has been the lack of commercial prospects. This generally implies the capacity for satellite servicing to prevent and repair failures. There have been numerous examples of spacecraft failures that could have benefitted from robotic intervention through on-orbit servicing [3]: (i) OAO-A2 lost its star sensor due to debris collision; (ii) OAO-C, Olympus-1 and ExoSat lost attitude control; (iii) NOAA-6 accidently vented its hydrazine incurring an uncontrolled tumble; (iv) Hipparcos was launched into the incorrect orbit due to a failure in its apogee kick engine; (v) ATS-6 suffered a thruster failure, etc. There is little doubt that robotic arms—which can also mount specialised or general tooling—offer a versatility that cannot be paralleled. Indeed, in 2020, the Mission Extension Vehicle (MEV-1) successfully attached to the retired Intelsat 901 communications satellite. It deployed an apogee engine probe which pulled the 901 so that its launch adapter ring pressed against three stanchions on the MEV. It was maneouvred back from a graveyard orbit into a geostationary orbit to provide an additional 5 years service to its nominal 18-year lifetime through the supply of power and propellant from the MEV. 

## 2. Space Salvage

An alternative approach to space debris mitigation is to recover the debris and, rather than de-orbiting it, exploit it as a resource—as salvage. This might be regarded as a more sustainable approach to space debris control. Only robotic manipulation is flexible enough to deal with both large and small debris unlike harpoons and nets which generate complex uncontrollable dynamic interactions between the robotic freeflyer, the target and the flexible umbilical connecting the two. This favours space debris mitigation through the deployment of freeflyer spacecraft mounted with dextrous manipulators which provide controlled interaction with the target [4]. Robot manipulators have been the workhorse of industrial applications for a range of tasks where precision positioning is required including machining, welding, sanding, spraying and assembly. For machining applications, parallel kinematic machines such as the six degree of freedom Stewart platform are unnecessary given that three or five degrees of freedom are sufficient and can offer high position accuracy [5]. However, it has been recommended that one degree of freedom redundancy above six degrees of freedom is included to compensate for joint failure [6]. The 75 kg Baxter with two seven degree-of-freedom arms is a new industrial standard which has a teach-and-follow facility. The arms are driven by series elastic actuators which give it high compliance. We propose that a minimum of two arms are required for grappling space debris targets. It is presumed that capture of defunct spacecraft will occur using either specialised tooling applied through the apogee thruster or at attachment points on the launch adaptor ring. We propose a bio-inspired freeflyer concept that specifically addresses the requirement for adaptability to a range of space debris sizes, offering a salvage solution that is robust to any orbital band. For robotic manipulation, there are three major manoeuvre requirements: (i) controlling freeflyer stability whilst manoeuvring the arms to grapple the target; (ii) manoeuvring the composite object once grappling is completed and then warehousing the captured assets (such as at the International Space Station); (iii) salvaging parts from the debris target for re-processing into new space assets. 

During the initial manoeuvre for grappling, there is dynamic coupling between the manipulator arm(s) and the spacecraft base on which it is mounted. The freefloating mode involves controlling the manipulator arm but allowing the spacecraft base attitude to be uncontrolled. There are a host of reasons why this is undesirable, most prominently being the existence of unpredictable dynamic singularities [7]. It has been proposed that a controlled floating mode be adopted that simultaneously controls the manipulator arm trajectory and the spacecraft attitude trajectory so the spacecraft attitude is altered controllably [8]. However, it is usually desirable that the spacecraft attitude remains fixed to maintain nominal pointing of antennas, solar arrays and sensors. For this reason, we suggest that the freeflying mode be adopted in which the spacecraft base is stabilised and the manipulator arm trajectory is controlled with respect to it [9]. This eliminates dynamic singularities. Furthermore, traditional rigid-body manipulator controllers such as the computed torque robotic controller [10] can be readily adapted to modal control to suppress vibrations by using a virtual rigid manipulator approach—this is achieved by replacing actual endpoint kinematic variables by those of a virtual rigid manipulator [11]. This approach, despite the increased degrees of freedom introduced by flexibility, allows the use of the smaller number of joint actuators to enforce tracking of the desired end effector trajectory. The feedback gains in the computed torque can be reduced by introducing feedforward torques learned through Gaussian process regression [12]. On the transition from phase (i) to phase (ii), there is the problem that there are limitations in the ability of traditional feedback control systems to deal with rapid complex dynamic responses whilst grappling the target, i.e., current techniques employed in space manipulators are insufficiently adaptive and robust to handle forces of interaction from widely varying space object geometries, sizes and manipulability [13]. Rapidly changing interaction forces during manipulation can introduce instabilities in the feedback control loop due to insufficient reactivity. This can be particularly acute if the payload dynamics are only partially known. It is crucial that robust adaptive robotic manipulation is developed to solve the problem of space debris mitigation. Phase (ii) is the conventional problem of orbital manoeuvring into different orbits through the consumption of fuel—we do not address this here. Force control issues will also be essential for phase (iii)— salvage is in fact an extension of on-orbit servicing which will involve a suite of complex operations involving the control of interaction forces between tooling and target. Indeed, salvage goes beyond servicing and repair of satellites to incorporate space manufacturing processes. 

The salvage and recycling of space debris involves producing feedstock for manufacturing new spacecraft such as standardised cubesat designs. The idea of salvaging spacecraft goes back to proposals for orbiting and refurbishing the shuttle external tank as a useful volume. Here, we are proposing salvaging space debris especially intact spacecraft as an approach to sustainability. Salvage recovers high quality materials and systems from intact but dysfunctional spacecraft. It is essential to recover everything to prevent debris creation. We propose recovering large decommissioned equipment items that can easily be separated and refurbished as a resource using powered tooling that can be exploited safely: (i) aluminium tankage, plates, radiators and frames—typically monolithic structures that can be removed and/or cut if necessary and re-used as-is; (ii) thermal blankets—typically on the spacecraft surface linked through standardised folds which should reduce cutting requirements but requires sophisticated handling; (iii) wiring harnesses—wound around the internal cavity of the spacecraft restrained by secondary brackets and cable ties that can be cut permitting wholesale removal of the harness; (iv) motors/gearing drives for deployment mechanisms and motorised pumps—motors and gears will require re-lubricating with silicone grease or dry lubrication; (v) solar array panels—solar cells may be refurbished using laser annealing; (vi) reaction/momentum wheels and gimbals—located internally and recovered intact for propellantless attitude control; (vii) payload instruments/attitude sensors—located externally and recovered intact (though external camera optics may be degraded due to radiation exposure, dust, etc. which may be rebuffed using abrasives); (viii) antennae/travelling wave tubes/transponders/radiofrequency electronics—located externally/internally as a module recovered intact for direct re-use. 

Aluminium foam structures are commonly adopted as the core between welded aluminium sheets for lightweight space structures and do not require heat-limited adhesives typical of common sandwich materials [14]. We assume that the majority of tasks to dismantle the target spacecraft involves similar tasks as employed during the Solar Maximum Repair Mission (1984) which comprised two main tasks: (i) orbital replacement exchange of a standardised externally-mounted attitude control module box through bolt manipulation (a variation on the peg-in-hole task) using standard power tools; (ii) replacement of an interior-mounted main electronics box that required manipulation of thermal blankets using specialised tooling [15]. Designing spacecraft for servicing involves the use of standardised modules (ORUs—orbital replacement units) with capture-compatible interfaces such as grapple pins, handrails, tether points, foot restraint sockets, standardised access doors, and makeable/breakable electrical/mechanical connectors. Standardised module exchange involves the manipulation of standard bolts, the preferred bolts being M8 and M10 hexagonal bolts with double head height. Captive fasteners should be employed, or the tooling should employ captive devices to prevent the loss of bolts as a further source of debris. Connector plugs should require no more than one single-handed turn to disconnect [16]. Standard power tools are automated threaded fastening systems with a typical torque range of 0.5–3.0 Nm. However, few spacecraft have been designed for servicing (the 1993 Hubble space telescope repair mission involved 150 types of tooling) necessitating substitution of standard tooling with adaptable tooling. 

For components that cannot be disassembled, robot manipulators with milling tools are essentially a development from CNC (computer numerically-controlled) machines employed for a wide range of manufacturing operations [17]. Laser machining is a subtractive manufacturing process in which a laser is employed to ablate material locally in cutting. Alternatively, NASA’s Universal Hand Tool (UHT) utilises electron beams at only 8 kV for welding or cutting thin metal sheets without producing dangerous X-rays. We do not recover onboard computers, batteries or propellant as these are likely to be in a depleted, unusable or dangerous state—an important exception to this are field programmable gate array (FPGA) processors which can be reprogrammed despite physical degradation (and for this reason have been proposed for employment on long-duration starship projects [18]). Thermal heat pipes represent an unknown factor—they have high utility but will require containment of the fluid medium rendering them a challenge for robotic handling. Excess aluminium from secondary structure may be melted by solar Fresnel lens and powdered for 3D printing by selective solar melting (in particular, for the production of solar sail segments [19] to provide propellantless propulsion to compensate for the lack of recovered propellant). Such solar sails may also be deployed as drag sails in low Earth orbit or orbit raising propulsors in geostationary orbit though this is disposal by traditional means. 

The left-overs once these bulk items have been stripped will be dominated by aluminium, lithium compounds from batteries and silicon and other minor materials in computer chips. The explosivity of lithium can be exploited to heat and melt the silumin-like alloy using solar Fresnel lenses which may then be powdered or drawn into wire. This requires considerably higher temperatures than aluminium smelting. Silumin alloy is a high-performance alloy used in high wear applications, but it is unclear what the effects of minor contaminants such as lithium (which may be readily excluded) and copper might be. These provide resources that can be 3D printed into any variety of joining structures to build new satellites in situ fitted with recovered panels, frames, components, etc. Additive manufacturing of complex net-shaped parts of polymer, metal, ceramic and composites has been proposed for microwave and millimetre-wave radiofrequency component manufacture in satellites [20]. The primary metrics for assessing the relative merits of 3D printing methods are dimensional accuracy, surface finish, build time and build cost [21,22,23]. Laser additive manufacturing is one approach to layer-by-layer construction though we propose a Fresnel lens-based approach. The only aspect that requires re-supply for new spacecraft is new computer chips and associated electronics from Earth (unless FPGA processors are recovered for their reconfigurability). An alternative approach is to grind mixed materials into a powder for pyrolytic/electrostatic/magnetic separation into its component materials. This would permit exploitation of 3D printing to print de novo cubesat constellations without the restrictions imposed by pre-existing plates, frames, etc. The most complex components of the spacecraft are computer chips which comprise aluminium metal strips, doped silicon semiconductor, silica insulation (especially in silicon-on-insulator technology), copper interconnects and a host of more exotic materials. Although Al can be separated through liquation at 660 °C, recovery of the other materials will be more problematic. Silica can be recrystallised and purified through zone refining but this is a complex process. This favours processing simplicity by reprocessing chips mixed with secondary structure in exploiting a silumin-type alloy as 3D printing feedstock without prior separation. 

Salvage is a sustainable form of disposal—it is rarely discussed in the context of active debris removal but it is entirely consistent with the growing sophistication of space missions. It would be far more cost-effective to convert these potentially valuable resources into high-utility commodities than to burn them up in the atmosphere or emplace them into graveyard orbits. It is also consistent with current developments in in-situ resource utilisation (ISRU) of the Moon and asteroids [24,25]—indeed, it shares much of the same fundamental technology of materials processing, e.g., complex assembly/disassembly manipulation, physicochemical purification, Fresnel lens pyrolysis, electrolytic processing, 3D printing, etc. Indeed, much of the equipment already in Earth orbit would have high value if transported to the Moon. Copper, so useful for electrical cabling, is extremely rare on the Moon yet miles and miles of wiring harness reside within defunct spacecraft in GEO and elsewhere. Commerce is a far more effective debris removal strategy than recommendations or regulations (which are ignored anyway). This solution will effectively convert a disaster into a bonanza. This effectively eliminates the dangers of debris re-entry and converts what is essentially waste into recycled assets. The key to this capability will be sophisticated robotic manipulation. 

## 3. Lessons from the Factory

Compliant manipulation is a fundamental requirement in manufacturing robotics. The importance of mechanical design in easing the complexity of control systems was illustrated by McGeer with a purely mechanical bipedal walking machine [26]. The RCC (remote centre of compliance) device is designed to ensure passive mechanical compliance in manipulator end effector behaviour during peg-in-chamfered hole (or screw which is essentially a helical peg) assembly tasks, the most common of assembly tasks encountered in manufacturing [27,28]. The phases of a peg-in-hole task are (i) gross motion approach; (ii) chamfer crossing; (iii) single-point contact; (iv) two-point contact; (v) final alignment. Maximum angular error is given by, $\theta_{\max}=\frac{(D-d)/D}{\mu }$ where *d* = peg diameter, *D* = hole diameter, *µ* = coefficient of friction. The RCC is mounted at the interface between the tool and the wrist mount and has a nominal lateral stiffness of 25 N/mm and rotational stiffness of 325 Nm/rad. Any peg-in-hole error displaces the axis of the peg with respect to the axis of the hole, thereby preventing jamming. It acts as a multi-axis “float” to allow mechanical linear and angular misalignments of up to 10–15% between parts by deflecting laterally and/or rotationally to permit assembly. Indeed, when the tool experiences a contact force or torque, the RCC deflects laterally and/or rotationally in proportion to the contact forces/torques with the RCC internal stiffness providing the constant of proportionality. The RCC as a mechanical device offers zero-delay response. The size range of RCC is limited, does not allow for high speed dynamics and requires chamfered holes. The instrumented RCC (IRCC) is based on the RCC but includes three strain gauges and three LED-detector pairs to provide active compliance across all three axes to cope with non-chamfered holes [29]. The RCC and IRCC provide a fixed stiffness and fixed location for the centre of compliance, limiting its use to specific scales of assembly operations. The variable RCC (VRCC) offers variable mechanical impedance and remote centre location through the addition of three motors for adaptive positioning with high precision [30]. These provide greater flexibility for variable peg-in-hole operations that are ubiquitous in assembly. There are detailed algorithms for computing the state of the peg-in-hole task with associated force measurements [31]. Exploratory guarded moves allow discovery-based behaviour to be implemented—indeed, most compliant exploratory tasks are in fact variations on the peg-in-hole task.

Designing and fabricating fixtures to flexibly secure workpieces for machining is an important aspect of the manufacturing process—indeed, the difference between manipulator grippers and dynamic fixtures is subtle. Robotic manipulators can exploit visual monitoring of automated microelectronic component assembly with actuated jigs [32]. Jigs and fixtures are mechanisms to impose structure into the work environment. They are devices to position, orient and constrain the workpiece to ensure fidelity of manufacturing operations which subject the workpiece to external forces and torques. The functional requirements of a fixture include location, constrained degrees of freedom, rigidity, repeatability, distortion and tolerances. Traditionally, fixtures are single purpose and designed for a specific part and require significant human involvement for setup and reconfiguration. There are certain design constraints to fixtures [33]: (i) form closure in which position/orientation wrenches are balanced to ensure workpiece stability to perturbations; (ii) accessibility/detachability subject to geometric constraints without interference; (iii) no deformation during fixture clamping. These constraints require kinematic, dynamic force and deformation analysis. There are four basic types of fixture—baseplate, locators, clamps and connections—and there are four major module fixturing interfaces—non-threaded hole, T-slot, dowel pin and reconfigurable system. There are 12 degrees of freedom to any workpiece (±x, ±y, ±z, ±R, ±P, ±Y). The 3:2:1 fixturing principle determines the locating points: (i) three support points are assigned to the first plane (restricting five degrees of freedom); (ii) two points are assigned to the second plane (restricting three degrees of freedom); (iii) one point is assigned to the third reference plane (restricting one degree of freedom), i.e., nine degrees of freedom are restricted by supports with the last three degrees of freedom restricted by clamps. Ideally, a single fixturing system must be automatic, flexible, adjustable and reconfigurable to accommodate different workpiece sizes and shapes. This will require the reconfigurable fixture to be comprised of standard modules which can be reconfigured robotically with the fixture design automatically determined by the requirements. An example fixturing system comprised four fixture modules mounted onto a baseplate including vertical supports that were fixed first followed by horizontal supports [34]. The supports guided the emplacement of the horizontal and vertical clamps. They were all based on a vertical threaded bolt with a pair of locking levers which could be manipulated by a single-handed manipulator. There were at least three vertical supports constraining the centre of gravity for maximum stability. The horizontal supports were emplaced as far apart as possible to impose kinematic constraints. The clamps permitted adjustment of clamping forces. End point surface geometries may be solid cone, hollow cone, flat face or spring-loaded bracket. Intelligent fixturing is enabled through sensors (position and force) to measure clamping positions and motors to actuate the fixtures. In addition, visual imagers may be used to plan fixture grasping. These are robotic fixtures with robot manipulators for loading and unloading of the fixtures. Pins can become solenoid-driven actuators. There are several approaches to fixing arbitrarily shaped parts—(i) modular fixtures, electromagnetic fixtures, and electro/magnetorheological fluid fixtures; (ii) adaptable fixtures (3D gripper); (iii) self-adapting fixtures (vices with movable jaws). These all have their limitations limiting their range of applicability and the requirement for positioning set up which is usually accomplished manually. The self-adapting jaw with pin arrays that mould to the geometry of the part provides high adaptability. Adaptive fixtures such as two or three-fingered adaptive fixtures are based on manipulator grippers. Compliance may be implemented through shape memory alloy wires and electrorheological fluids, e.g., [35]. Finally, fixtureless assembly adopts robotic tools and grippers with the use of fixtures. Grippers can form a hook grip, a scissor grip, multi-fingered chuck, squeeze grip and multiple geometric grips through its capability to mould to many geometries.

## 4. Biomimetic Approaches to Robot Manipulation

Our goal is the replicate human level manipulation capabilities—it would appear appropriate then to examine human manipulation from a biomimetic perspective. The implementation of robust human-like manipulation offers the prospect for a wide range of applications involving robots dealing adaptively and safely with the real world. The problem in introducing robots into the wider world has been not their intelligence but their ability to physically interact with the world. Robust adaptive manipulation is the key to converting space junk into salvageable assets for re-use. The key facets to bio-inspired approaches to engineering are robustness and adaptability [36]. We believe that biomimetic approaches can provide this capability—to that end, we review biomimetic approaches to robotic manipulation emphasising the central role played by manipulator control systems. The Gibsonian theory of affordances states that there are ecological laws which relate an organism to its environment, the latter being an essential component to cognition [37,38]. Affordances are the potentials for action afforded by the properties of the environment (objects, events and locations) relative to the agent. The effectors are then actuated to realize a specific affordance. Hence, categorisation is not purely perceptual, but is sensorimotor. 

Sensorimotor control is the primary function of the brain for which several strategies are employed [39]: (i) sensorimotor planning, learning and control, (ii) optimal feedback control, (iii) impedance control, (iv) predictive control, and (v) Bayesian inferencing. The central nervous system is hierarchical at three levels—spinal cord, brainstem and cerebral cortex—and motor planning, learning and control activate multiple regions of the human brain [40,41] linked through persistent neural activity [42]: (i) prefrontal cortex especially the dorsolateral prefrontal cortex indicating working memory; (ii) left primary motor cortex indicating visuomotor skill learning; (iii) lateral premotor cortex indicating visuomotor association; (iv) supplementary motor area indicating motor sequence planning; (v) cerebellum indicating internal motor feedback control based on error; (vi) superior posterior parietal cortex indicating visuospatial processing; (vii) anterior-inferior parietal cortex indicating sensory feedback processing; (viii) basal ganglia indicating motor action selection. It has been proposed that different motor areas of the brain are characterised by their differing learning algorithms [43]. The cerebellum is characterised by a highly ordered structure, the basal ganglia by its multiple inhibitory pathways, and the neocortex by its six-layer structure with the cerebellum and basal ganglia being reciprocally connected to the cerebral cortex. The cerebellum implements supervised learning based on error vectors to form internal models of the environment, the basal ganglia implements reinforcement learning based on scalar rewards to perform action selection through the evaluation process, and the cerebral cortex implements unsupervised learning with teaching signals to statistically estimate the state of the environment [44]. The motor cortex acts as a control system using visual feedback but acting in concert with a feedforward dynamics model of the musculoskeletal system in the cerebellum. The emulator (forward model) must be capable of adapting to changing circumstances such as limb growth, musculature changes over time, changes in sensory sensitivity, etc. Continuous self-modelling is accomplished using sensorimotor relationships to infer its own state [45]. The robot continuously learns its morphological structure which permits it to adapt to morphological changes by restructuring its self-model. In particular, it invokes actions to generate sensory data that may be used to train a predictive neural network model. Cortical neurons exhibit Hebbian type adaptability in which they are active when presynaptic input and postsynaptic response are associated. It appears that an indirect adaptive control system is implemented in the motor cortex whereby the adaptive controller is implemented through the estimation of plant parameters rather than directly through input–output signals. These are integrated into a goal-oriented system for generating human motor behaviour.

## 5. The Nature of Sensorimotor Control

In a robotic or biological manipulator, a set of actuators (muscles or motors) at the joints are driven to effect cartesian movement of the end effector (hand or tool). Motor control relates actions on the environment to their sensory effects through a transformation function. Any planned end effector trajectory for a multi-joint arm must undergo sensorimotor transformation into joint coordinates. In the human brain, the primary motor cortex and supplementary motor area encode adaptation of kinematic-dynamic transformations of movements. Voluntary movement requires three main computational processes to be implemented in the brain: (i) determination of cartesian trajectory in visual coordinates; (ii) transformation of visual coordinates into body coordinates in which proprioceptive feedback occurs (within the association cortex); (iii) the cartesian trajectory in body coordinates θ^d^ is converted into the generation of motor commands τ (within motor cortex) to the muscles through the spinal cord. Internal models are used as neural models of aspects of the sensorimotor loop including interaction with the environment to predict and track motor behaviour. The primary motor cortex (M1) implements inverse models that convert desired end effector cartesian trajectories into patterns of muscle contractions at the joints (output), i.e., motor commands. These coordinate transformations between external world coordinates to joint/muscle coordinates appear to be implemented between M1 and the ventral premotor cortex (PMV) [46]. The first mapping that must be achieved is the nonlinear transformation of task coordinates of the end effector q in terms of joint coordinates θ:

(1) q=f(θ)

where *f*(*θ*) = 4 × 4 Denavit–Hartenburg matrix (an SE(3) Lie group). This may be differentiated to yield cartesian velocities relation to joint velocities through the Jacobian matrix *J*(*θ*):

(2) $$\dot{q}=J\left(\theta \right)\dot{\theta }$$

where *J*(*θ*) = 6xn Jacobian matrix for n degrees of freedom. From virtual work arguments, the transpose of the Jacobian relates joint torques *τ* to cartesian end effector forces *F*:

(3) $$\tau =J^{T}F$$

If the manipulator is kinematically redundant (i.e., *n* > 6), the Moore–Penrose pseudoinverse is the generalised inverse:

(4) $$J^{*}=J^{T}{\left(JJ^{T}\right)}^{-1}$$

The inverse dynamic model for a robotic manipulator is given by the Lagrange–Euler equations describing the output torque τ required to realise the observable kinematic state of the manipulator joints ${\left(\theta ,\dot{\theta },\ddot{\theta }\right)}^{T}$:

(5) $$\tau =D\left(\theta \right)\ddot{\theta }+C\left(\theta ,\dot{\theta }\right)+G\left(\theta \right)+J^{T}F$$

where *D*(*θ*) = inertia parameter of the manipulator, $C\left(\theta ,\dot{\theta }\right)$ = Coriolis and centrifugal parameter of the manipulator, *G*(*θ*) = gravitational parameter of the manipulator (in the case of space manipulators, this term vanishes), *F* = external force at the manipulator end effector, *J* = Jacobian matrix. The adaptive finite impulse response (FIR) filter may be used to approximate the inverse dynamic model of a process through mean square error minimisation [47]. Several regions of the brain project into motor area M1 providing feedback signals from the primary somatosensory cortex, posterior area 5 and from the thalamus via the cerebellum. In feedback control, the actual trajectory is compared with the desired trajectory thereby defining the tracking error. This error is fed back to the motor command system to permit adjustments to reduce this error. Control systems exploit inverse models to compute the desired motor action required to achieve the desired effects on the environment (such as a desired trajectory). The feedback controller computes the motor command based on the error between the desired and estimated states. The motor command is the sum of the feedback controller command and the inverse model output. Inverse internal modelling of the kinematics and dynamics of motion is similar to adaptive sliding control [48]. The internal model constitutes an observer and essentially represents the reference model employed in adaptive controllers. An inverse model may be generated by inverting a forward model neural network representing the causal process of the plant [49]. Forward models define the causal relationship between the torque inputs and the outputs states of the motor trajectory (position/velocity) and the sensory states given these estimated states. The parietal cortex is concerned with visual control of hand movements—it requires 135–290 ms to process visual feedback. It computes the error between the desired Cartesian position and the current Cartesian position, the latter computed from proprioceptive feedback measurements from muscle spindles [50]. This requires an efference copy of the motor commands to create a feedforward compensation model. An efference copy (corollary discharge) of these motor commands is passed to an emulator that models the input-output behaviour of the musculoskeletal system. A hierarchical neural network model can emulate the function of the motor cortex [51]. There is an error between the actual motor patterns *θ* (and $\dot{\theta }$) measured by proprioceptors and the commanded motor patterns *τ* from the motor cortex which is fed back as $\theta^{d}-\theta$ with a time delay of 40–60 ms. A forward dynamics model of the musculoskeletal system resides within the spinocerebellum-magnocellular red nucleus system. The forward model receives feedback from the proprioceptors *θ* and an afferent copy of the motor command *τ*. Thus, the forward model takes the motor command *τ* as input and outputs the predicted trajectory θ*. The forward model predicts the movement θ* which is used in conjunction with the motor command *τ* to compute a predicted error $\theta^{d}-\theta^{*}$ which is transmitted to the motor cortex with a much shorter time delay of 10–20 ms. The forward model predicts the sensory consequences of the motor commands. This top-down prediction is based on a statistical generative model of the causal structure of the world learned through input-output relations. In humans, this forward model of the musculoskeletal system has been learned since the earliest motor babbling that begins after birth. In athletes, it is refined through physical training who refer to it as muscle memory. An inverse dynamics model of the musculoskeletal system resides within the cerebrocerebellum-parvocellular red nucleus system—it does not receive sensory inputs. The inverse dynamics model has the desired trajectory θ^d^ as input from which it computes motor commands τ as output. The inverse dynamics model must learn to match the forward model to generate accurate motor commands τ in order to compensate for variable external forces. The integral forward model paradigm places the forward model at the core of all perception–action processes—this is the basis for the integral forward model in which sensor and motor functions are fully integrated [52]. Forward models are employed to make predictions that provide top-down expectations to incoming bottom-up sensory information. Mismatch generates a prediction error that induces refinement of expectations.

## 6. The Role of Optimal Feedback Control

The primary motor cortex (M1) adopts optimal control strategies for voluntary movement [53]. Indeed, activity in the motor cortex predicts movement before it occurs—such movement requires projection through the pyramidal tract into the basal ganglia and brainstem [54]. The multilayer perceptron (MLP) can implement a nonlinear plant model controlled by a generalised predictive control algorithm with Newton–Raphson minimisation of a cost function [55]:

(6) $$w_{i+1}=w_{i}-{\left(\frac{\partial^{2}J_{i}}{\partial w_{i}^{2}}\right)}^{-1}\frac{\partial J_{i}}{\partial w_{i}}$$

where *J* = cost function. There are an enormous number of possible muscle activation patterns that can yield a given hand trajectory. The two-thirds power law relation states that the angular velocity of the end effector is proportional to path curvature to the two-thirds power [56], $\left|w\left(t\right)\right|=\alpha C{\left(t\right)}^{\frac{2}{3}}$ where *w* = end effector angular velocity, *α* = velocity gain factor = 0.66 empirically, *C*(*t*) = path trajectory curvature. It appears that *α* correlates with the average movement velocity within each movement segment. Optimal control selects the optimal plan according to minimum jerk consistent with the two-thirds power law with the two-thirds exponent arising from the viscoelastic properties of the muscles [57]. The cost function serves to select straight-line end effector pathways with bell-shaped velocity profiles from an effectively infinite set of possible muscle activations—the 600 muscles of the human body for controlling over 200 joints yield 2^600^ possible muscle activations (not considering degrees of activation). This is far beyond the capacity of simple look-up table associations, yet stereotypical movement patterns suggest that optimal control is applied with cost constraints imposed to reduce the selection task. The cost may be subjected one of two constraints. The cost to be minimised is the rate of change of acceleration of the hand (jerk):

(7) $$C=\frac{1}{2}\int_{0}^{T}{\left(\frac{d^{3}x}{dt^{3}}\right)}^{2}+{\left(\frac{d^{3}y}{dt^{3}}\right)}^{2}dt$$

where (*x*,*y*) = hand position, *T* = duration. This is a kinematic trajectory. Alternatively, the cost to be minimised is the rate of change of torque at the joints (torque change):

(8) $$C=\frac{1}{2}\int_{0}^{T}\sum_{i=1}^{n}{\left(\frac{d\tau_{i}}{dt}\right)}^{2}dt$$

where *τ* = joint torque. This is a dynamic trajectory. Although the two cost functions are related through the relationship of torque to acceleration, trajectories are planned in kinematic rather than dynamic coordinates [58]. Hence, minimum jerk accounts for straight line paths with bell-shaped velocity profiles. This yields the general form of end effector trajectory given by a fifth order polynomial:

(9) $$x\left(t\right)=a_{0}+a_{1}t+a_{2}t^{2}+a_{3}t^{3}+a_{4}t^{4}+a_{5}t^{5}$$

where *a*_i_ = parameters constrained by boundary conditions on position, velocity and acceleration. Muscle movements and limb trajectories are characterised by spatiotemporal constraints where the shape of the τ function is given by [59] $\tau (t)=\frac{1}{2}\left(t-\frac{T^{2}}{t}\right)$ where *τ* = time to complete movement, *T* = total movement time, *t* = elapsed time from the start of the movement. This gives a constant acceleration trajectory. Parabolic paths yield both a two-thirds power law and a minimum jerk trajectory. The sensorimotor system is subject to optimal feedback control according to minimisation of the time integral of a cost function [60]. The minimal intervention principle is a property of optimal control in which deviations from nominal trajectory due to uncertainty are corrected only when they interfere with task performance [61]. Hence, the appropriate cost function to be minimised is total expected cost. This provides constraints on the selection of internal models—a learned internal model obviates the search for the muscle activations problem [62]. The internal model involves two transformations: muscle motor commands to Cartesian end effector state (forward model), and Cartesian end effector state to muscle motor commands (inverse model). The forward model predicts the expected output behaviour of a muscle input command. This enables the forward model to estimate the current position/velocity of the limb in the presence of feedback delays.

## 7. Why Predictive Control?

The first function performed by the brain is perception. Perception involves a cycle of top-down processing in which a top-down feedforward model predicts perceptual expectations to guide bottom-up sensory processing of perceptual data fed back from sensors, i.e., perception is indirect and an internal model constitutes the brain’s current hypothesis of the world. Any prediction error requires either actions to alter sensory data to conform better to the internal model or the model must be adapted to reduce this error. Prediction is a key general neural computation in the human brain and indeed predictive errors drive neural processes [63]—prediction is the basis for hierarchical causal state sequencing of behaviour [64]. Neural learning is fundamentally based on performance error as the difference between the actual output compared to the desired output. Learning is based on the predicted contingency of events based on likelihood rather than simple contiguous association. Generalisation through learning of an input-output model is a predictive process that predicts future outputs on the basis of past observations (x_i_,y_i_). Current observations reduce the posterior distribution’s entropy from its prior value. Classical and operant conditioning are thus predictive processes in modelling the world’s past behaviour. Conditioning provides the mechanism for associative learning of feedforward predictive internal models of social situations [65]. These CS-US predictive models overcome the problem of time delays in feedback control through memory. Control is effected across a communications channel and its noise characteristics impose an information rate capacity limit R on feedback [66,67]. Predictability provides a probabilistic measure of Kolmogorov–Chaitin complexity (minimum program length that simulates an observed sequence of data) [68,69]. Predictive information is given by mutual information between the past and the future [70]: $I_{pred}=log_{2}\left(\frac{p(x_{t>t_{0}}|x_{t<t_{0}})}{p\left(x_{t>t_{0}}\right)}\right)$. For a Markov process, observation at *t*_i_ depends only on events at the previous time step *t*_i-1_: $I_{pred}=log_{2}\left(\frac{p(x_{i}|x_{i-1})}{p\left(x_{i}\right)}\right)$. The minimum description length is equivalent to Bayesian estimation which updates a prior model with new observations. If entropy is defined as the negative logarithm of Bayesian evidence, the brain maximizes Bayesian evidence through predictive coding. Entropy corresponds to the prediction error generated by comparison of top-down predictions (expectations) and bottom-up sensory evidence. The prediction error is passed up the processing chain to the higher processing areas to update the model. This effectively implements a gradient descent on the sum of squared prediction errors. The brain acts as a Helmholtz inferencing engine in which a generative model is used to predict sensory effects of hidden neural states [71]. Helmholtz machines are artificial neural networks that learn real world data by minimising their generative free energy through alternating wake and sleep cycles. The Bayesian framework assesses contextual environmental information to select a prior forward model which generates a prediction error and allows selection of the forward-inverse pair appropriate to the situation. A forward model is thus a prior prediction updated by sensory measurements [72,73]. The prior forward model generates predictions to minimise those errors. Learning minimises a function of the prediction error subject to a priori constraints usually through gradient descent. Minimisation of the predictive error is an expression of the infomax principle.

Biological neural feedback circuits are slow with only small gains so neural control systems exhibit large feedback delays. Time delays in negative feedback systems $\frac{G}{1+G}$ can cause positive feedback $\frac{G}{1-G}$ due to phase shifts thereby causing instability because responsive action is elicited only when sensors have detected a deviation and fed back that deviation. Delayed feedback imposes an upper bound on feedforward capacity C. For the rapid monosynaptic spinal reflex, the delays are short at 20–50 ms to which muscular force generation adds another 25 ms. Visual feedback is delayed by 100–300 ms due to sensory transduction, visual neural processing times and neural transmission times. This equates to the onset latency of neurons of the inferotemporal cortex implying that image processing involves a single feedforward pass through the visual pathways. The total trajectory movement time is 150–500 ms in duration so time delays represent a major fraction of the movement duration. The Stroop effect is a cognitive interference effect due to mismatching stimuli that shows differing processing speeds. It is most commonly demonstrated in mismatching the name of a colour and the colour of its print, e.g., “green” printed in a red colour. The brain can read words automatically which is faster than its processing of colour. The effect of proportional feedback control systems with time delays is a growing oscillatory response that becomes chaotic and destructive - small changes in applied torques generate divergent sensed forces on contact with stiff environments ≈10^4^–10^6^ N/m which cannot in general be compensated for by closed loop force control. As feedback delay increases, delayed feedback capacity R decreases to a limit of zero feedback at infinite delay, i.e., delayed feedback capacity R approaches the feedforward capacity C as the delay increases [74]. The forward model predicts the sensory consequences of motor commands with a much shorter time delay of 10–20 ms. Feedforward control is a form of moving average filter while feedback implements an autorecursive filter which is determined by the poles and zeros of the transfer function. Rapid movements such as fast eye movements cannot be executed using feedback control as feedback loops are too slow with delays of 150–250 ms compared with movement durations of 150–500 ms—they must be implemented using feedforward controllers.

## 8. Models—Backwards and Forwards

Large delays in neural feedback signals from sensors make pure feedback strategies implausible so predictive feedforward control is necessary with feedback being used to correct the trajectory. Two copies of a motor command are generated by the inverse model, the efference copy being passed to the forward model to simulate the expected sensory consequences which are compared with actual sensory feedback. The forward predictive model is essential for skilled motor behaviour—it models how the motor system responds to motor commands. In the forward model, motor commands are input to the forward model and transformed into their sensory consequences as the output—forward models model the causal relationship between input actions and their effects on the environment as measured by the sensors. The forward internal model acts as a simulator of the body and its interaction with the environment, i.e., it constitutes a predictor. The forward dynamic model of a robotic manipulator is given by:

(10) $$\ddot{\theta }=D^{-1}\left(\theta \right)\left[\tau -C\left(\theta ,\dot{\theta }\right)-G\left(\theta \right)\right]$$

Joint acceleration $\ddot{\theta }$ may be integrated to yield joint rate $\dot{\theta }$ and joint rotation *θ* as the predicted output sensory states for torque input τ. Rather than actual accelerations, it has been suggested that desired accelerations may be employed to train these models [75]. The forward dynamic model imitates the body’s musculature which generates a predicted trajectory output from an efference copy of input motor commands [76]. Feedforward control thus uses a model of the plant process to anticipate its response to disturbances to compensate for time delays [77]. The predicted trajectory output may be fed to the input of the feedback controller to compensate for time delays. Forward models adapt 7.5 times more quickly than inverse models alone [78]. This forward predictive control scheme has been proposed as a model of cerebellar learning from proprioceptive feedback from muscle spindles and Golgi apparatus which measure muscle stretch. The forward model may be implemented as a neural network function approximator to the forward dynamics. This may be represented as a look-up table with weights learned from input-output pairs of visuomotor training data, e.g., CMAC (cerebellar model articulation controller) [79]. CMAC has been applied as lookup tables to reproduce input-output functions defined by the kinematics of a robotic manipulator [80]. CMAC has been applied to the grasping control of a robotic manipulator using CCD camera images transposed to object locations that were passed to a conventional robot controller [81]. The look-up table representation is not consistent with biology however —it appears that motor adaptation does not involve the composition of look-up tables rather than the forming a full and adaptable model which can extrapolate beyond the initial training data [82]. The Marr–Albus–Ito theory of cerebellar function represents a motor learning system similar to a multilayer perceptron [83]. Biology favours Gaussian radial basis function network representation [84] so the feedforward model may be implemented as a radial basis function network as a biomimetic representation [85]. Without calibration from actual sensory feedback, forward models will accumulate errors. The combination of feedforward (exploration) and feedback (exploitation) control provides an efficient approach to control systems. It is apparent that predictive forward models are learned prior to inverse models for the application of control [78]. 

**Figure 1:**
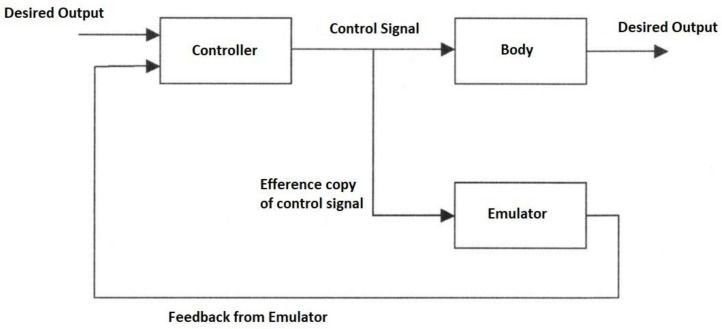
Forward model control configuration with efference copy.

The forward model can use an efference copy of the motor command as input to cancel the sensory effects of movement (Figure 1). 

The same process cancels the effects of self-motion on the senses to distinguish it from environmental effects (e.g., self-tickling). For each forward model, there is a paired inverse model to generate the required motor command for that context cued by sensory signals. In motor control, the full internal model comprises a paired set of forward and inverse models requiring two network pathways through an inverse model and forward model, respectively, with the latter acting as a predictor. The feedback controller converts desired effects into motor commands while the feedforward predictor converts motor commands into expected sensory consequences. The feedforward and feedback components interact continuously by combining both efferent and afferent signals in the forward model, i.e., sensory feedback is essential for the forward model [86]. The learning rule such as the delta rule of a neural network is similar to a model reference adaptation law and learns the inverse dynamic model [87]. The feedforward neural model can be used as a nonlinear internal model for a model reference adaptive controller [88]. The repertoire of motor skills requires multiple different internal models of smaller scope than a single large monolithic model can accommodate. Multiple paired forward and inverse models are necessary to cope with the large number of kinematic-dynamic situations that can occur. Hence, the MOSAIC (modular selection and identification for control) model proposes multiple pairs of forward predictor and inverse controller models to represent different motor behaviours [89,90]. This modular approach employs multiple tightly coupled inverse/forward model pairs for generating motor behaviour under widely disparate situations—32 inverse model primitives can yield 2^32^ = 10^10^ behavioural combinations [91]. A multitude of such modular paired forward-inverse models exist for different environmental conditions. Each predictor represents a different hypothesis test to determine which context is most appropriate with the smallest prediction error. The current context determines the selection of the predictor–controller pair. Rather than hard switching, modular forward-inverse model pairs are selected with weights. The set of sensory prediction errors from the forward models determine the probabilities that weight the outputs of the paired controllers—the combined output is the weighted sum of the outputs of the individual controllers. Multiple models may be active simultaneously whose outputs may be summed vectorially to construct more complex behaviours. The mixture-of-experts is a divide-and-conquer strategy reducing complex goals into subgoals that are selected through a gating mechanism. The mixture-of-experts approach is a statement of Ashby’s principle of requisite variety [92]. Adaptability ensures that functionality is retained in the face of environmental perturbations—it does so by monitoring the environment and adjusting its internal parameters in response to maintain its behaviour. As the complexity of the environment increases, a greater diversity of more specialised internal control systems is required. Bayesian gating selection is accomplished from the likelihood of the forward-inverse pair with minimum prediction error $\left|x_{i}-{\bar{x}}_{i}\right|$:

(11) $$p_{i}\left(x_{i}|w_{i},y_{i},i\right)=\frac{1}{\sqrt{2\pi \sigma^{2}}}exp\left(-{\left|x_{i}-{\bar{x}}_{i}\right|}^{2}/2\sigma^{2}\right)$$

where *σ* = scaling constant. The prior probability for each sensory context *y_i_* is given by $\pi_{i}=f\left(w_{i},x_{i},y_{i}\right)$ where *f*(.) = forward model approximator (nominally, a neural network), *w_i_* = forward model weight parameters. Bayes theorem multiplies this likelihood with the prior followed by normalisation to generate posterior probabilities:

(12) $$P_{i}=\frac{\pi_{i}p_{i}}{\sum_{j}^{n}\pi_{ij}p_{ij}}$$

This soft-max function transforms errors through the exponential function which is then normalised to form a “responsibility” predictor. The posterior probability is used to train the predictive forward model to ensure that priors approach posteriors. Selection between expert modules may be learned to partition the input space into different forward model regions. A gradient descent rule estimates the probability of the suitability of each expert [91]:

(13) $$\Delta w_{i}=\eta P_{i}\frac{df{\left(.\right)}_{i}}{dw_{i}}\left(x_{i}-{\bar{x}}_{i}\right)$$

Alternatively, expectation maximisation such as the Baum–Welch algorithm as used in hidden Markov models (which are Kalman filters with discrete hidden states) offers superior performance to gradient descent. The inverse model paired with the selected forward model is then computed to implement the controller. The paired inverse model $y_{i}=f^{\prime }\left({w^{\prime }}_{i},x_{i+1},x_{i}\right)$ generates the required motor command through the same weighting [93]:

(14) $$y_{i}=\overset{n}{\underset{i=1}{\sum }}P_{i}y_{i}=\overset{n}{\underset{i}{\sum }}f^{\prime }\left({w^{\prime }}_{i},x_{i+1},x_{i}\right)$$

Similarly, learning of the inverse models is weighted:

(15) $$\Delta {w^{\prime }}_{i}=\eta P_{i}\frac{df^{\prime }\left(.\right)}{d{w^{\prime }}_{i}}\left(y_{i}-{\bar{y}}_{i}\right)\approx \eta \frac{dy_{i}}{d{w^{\prime }}_{i}}P_{i}y_{fb}$$

Prediction errors from the forward model are thus used by the inverse model to generate muscle contractions to generate actions on the world. Prediction errors can also be used to update the predictive forward model by comparing the actual and predicted sensory data from a motor command.

Any action sequence may be represented as a set of schemas organised hierarchically. The highest schema activates component schemas representing units of movement comprising the specific action sequence [94]. A motor schema is an encapsulated control system module with neural maps to define spatial-temporal control actions of muscles according to proprioceptive feedback signals [95]. Motor schemas appear to be represented in cortical areas 6 and 7a. The forward model constitutes a body schema representation that maps its sensorimotor behaviour for simulating actions without motor execution [96]. This emphasises forward and inverse kinematic transforms that can be updated through learning by recognising the correlation between visual images, proprioceptive and tactile feedback and motor joint commands. A Bayesian network model of manipulator kinematic structure where nodes represent body part poses has been shown to learn and represent the forward kinematic structure $p(q_{1},\ldots ,q_{n}|\theta_{1},\ldots ,\theta_{m})$ and inverse kinematic structure $p(\theta_{1},\ldots ,\theta_{m}|q_{1},\ldots ,q_{n})$ of a robotic manipulator [97]. The phantom limb syndrome in amputees, both congenital and subsequent, is due to neural network representation in the somatosensory cortex and connected areas with a genetic origin that is modified by sensory experience [98]. This is part of the body schema that emulates the human body. We can exploit tools to extend our senses to detect objects as if the tools were parts of our body [99]. Skilled tool use demonstrates that forward models are adaptively trained using prediction errors [100]. This sensory embodiment is enabled by the learning capacity of predictor–controller models of Euler–Bernoulli vibrating beams in the cerebellum. The weight of an object to be grasped must be predicted to determine the required grip force 150 ms prior to contact [101]—it is estimated as the weighted mean force from previous trials, i.e., a predictive model [102]. 

Grasping is an object-oriented action that requires an object’s size and shape be transformed into a pattern of finger movements (grip)—there is evidence that these processes are performed in the parietal lobe [103]. The fingers begin to pre-shape during large scale movements of the hands whereby the fingers straighten to open the hand followed by closure of the grip until it matches the object size. Such a process may be emulated by perceptual and motor schemas, i.e., a pre-shape schema selects the finger configuration prior to grasping. The Kalman filter has been applied to emulate synchronised human arm and finger movements prior to grasping [104]. Fingers open in a pre-shape configuration halfway through the arm reach. The two movements of finger shaping and arm reaching are synchronised and are determined by the object size and location. Both the ventral “what” visual pathway and the dorsal “where” visual pathway are required to transform visual information into motor acts [105,106]. In active vision, the motor system is a fundamental part of the visual system by orienting the visual field. Nevertheless, visual processing is inherently slow due to high computational resource requirements of processing images. Perception is predictive, illustrated by the size–weight illusion that a small object with the same weight as a large object is “felt” to be heavier. It appears that the F5 neurons of the premotor cortex involved in grasping are selective for different types of hand prehension—85% of grasping neurons are selective to one of two types of grip (precision grip and power/prehension grip). The power grip is relatively invariant involving the enveloping of the object but the precision grip is more complex with a variety of finger/hand configurations. There is also specificity in F5 neurons for different finger configurations suggesting the validity of the motor schema model. Prehensile gripping allows the holding of an object in a controlled state relative to the hand and the application of sufficient force on the object to hold it stationary. It imposes constraints through contact via structural and frictional effects. The grip force must exceed the slip ratio though it is preferred to minimise reliance on friction in favour of geometric closure. A condition for force closure is that the grip Jacobian G contains the null space of wrenches w_i_ from contact forces such that $\sum w_{i}=0$. Adaptive gripping requires tactile feedback [107]. Feedforward predictive models in conjunction with cutaneous sensory feedback are instrumental in adapting grip forces to different object shapes, weight and frictional surfaces [108]. The feedforward component provides rapid adaptability through prediction rather than just reactivity through pure cutaneous feedback. It is the combination of predictive feedforward and reflexive feedback that yields skilled manipulation. Both the cerebellum and cutaneous feedback are critical for the formation of grip force predictions, the latter being essential for detecting slipping. Predictive models provide the basis for haptic grasping indicated by preadaptations to learn complex motor skills [109]. Anticipatory grip-to-load force ratios precede arm movements and more generally, prediction always precedes movement. Hence, when lifting an unknown object, multiple forward models constructed from prior experience are active generating sensory predictions. The forward model that generates the lowest prediction error is selected activating its paired inverse model-based controller. A forward kinematic model of a robotic hand and arm has been learned through its own exploratory motions observed by a camera based on visual servoing [110]. Continuous self-modelling by inferring its own structure from sensorimotor causal relationships provides the ability to engage in autonomous compensatory behaviours due to injury [45]. Forward models are thus essential to for robust behaviour in animals.

## 9. Role of the Cerebellum

**Figure 2:**
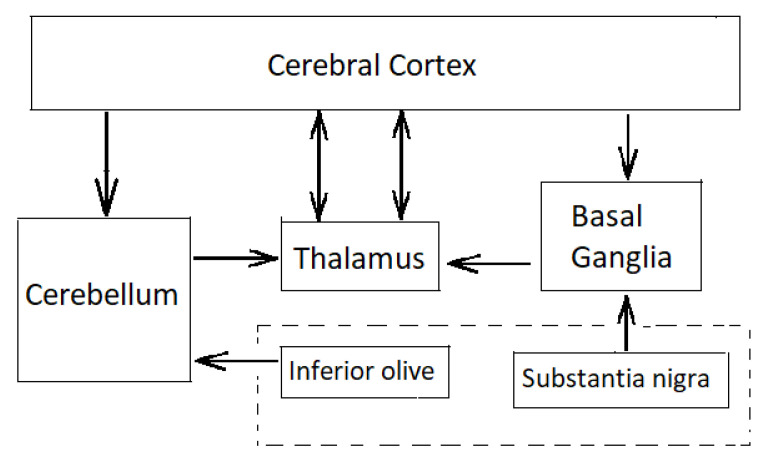
Cerebellar connections through neighbouring neural structures.

The cerebellum is one of the most widely connected structures of the brain with connections into all the major systems of the brain (Figure 2). 

The cerebellum implements fine motor coordination, balance, motor timing and motor learning, particularly in initiating and coordinating smooth fine-scale movements [111,112]. Cerebellar dysfunction has been attributed as the cause of tremor ataxia involving loss of motor coordination. The cerebellum comprises four major parts with a highly regular architecture—flocculus, vermis, intermediate hemisphere and lateral hemisphere. With 1.6 × 10^10^ neurons in a well-ordered architecture, it performs the same computation with different modules projecting widely into different parts of the brain as well as the motor cortex. The cerebellum possesses the largest number of neurons than any other structure in the human brain: it comprises rows of 10 M Purkinje cells, each receiving around 200,000 synapses from parallel fibre inputs from proprioceptors and climbing fibres carrying error signals. The 200,000 parallel fibres fire at a rate of around 60 Hz to a single climbing fibre that fire at a rate of just 1–2 Hz converging onto each Purkinje cell. Lesions of the cerebellum cause disruptions to the coordination of limb movement such as jerky movements, poor accuracy, poor timing, tremors, etc. implicating it in the regulation of movement. According to this theory, the cerebellum serves to coordinate the timing of the elements of muscular movement rather than the muscle movements themselves [113]. It achieves this through the modulation of gain control parameters. Inherent in this hypothesis is the existence of a somatotopic map of the body within the three cerebellar nuclei. Each cerebellar nucleus controls a different mode of body movement. Parallel fibres linking Purkinje cells somatotopically encode and control specific combinations of the body’s musculature. Purkinje cells linked by parallel fibres project onto cerebellar nuclei imposing different control modes to generate coordinated movement. These muscular pattern synergies control and coordinate different parts of the body. However, it is reckoned that the cerebellum has a far more central role to play in motor control. 

**Figure 3:**
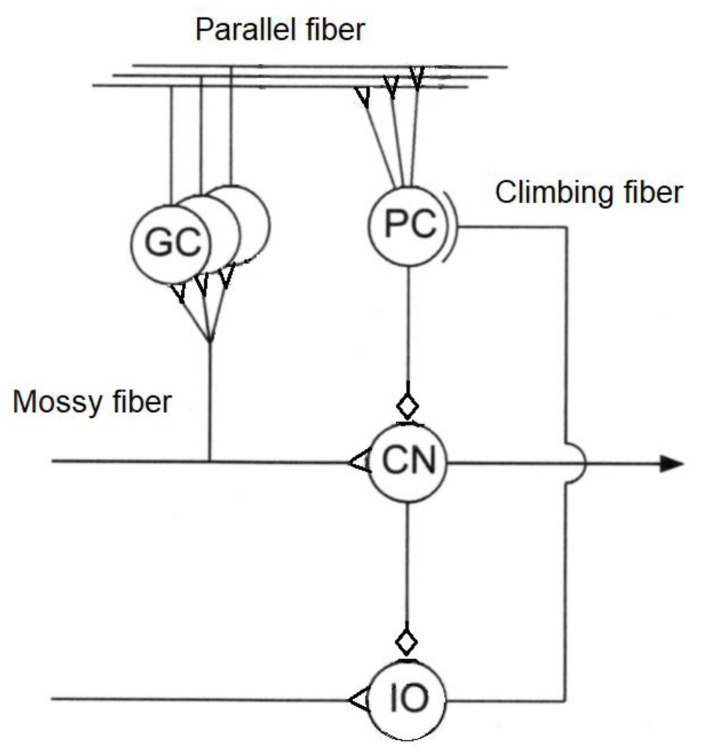
Simplified representation of the cerebellum’s functional structure (GC = granule cell, PC = Purkinje cell, CN = cerebellar nuclei, IO = inferior olive)—arrows indicate excitatory connections and diamonds indicate inhibitory connections.

The cerebellum acts as an associative memory between input patterns on granule cells and output patterns on the Purkinje cell patterns (Figure 3). 

Cerebellar input is based on two input channels—parallel fibres from mossy fibres and climbing fibres from olivary neurons. Output from the granule cells—parallel fibres—converge onto Purkinje cells with climbing fibres from the inferior olive. Synapses of parallel fibres connecting granule cells to Purkinje cells are modified by inputs from climbing fibres from the inferior olive to the Purkinje cells. The climbing fibre synapses onto the Purkinje cells reside in the cerebellar flocculus. As granule cells form a recurrent inhibitory network with Golgi cells, they represent a recurrent circuit. The cerebellum comprises a feedforward circuit in which inputs are converted into the output of Purkinje neurons that implement the predictor model. Its feedforward structure comprises a divergence of mossy fibres onto a massive number of granule cells that converge back onto Purkinje cells, i.e., it is highly integrative [114]. Granule cells are the most numerous neurons at around 10^10^–10^11^ in the human brain—the ratio of granule cells to Purkinje cells is around 2000:1. Granule cells are small glutamatergic cells which receive feedback inhibition from Golgi cells. Each granule cell has 3–5 dendrites and its axon bifurcates into parallel fibres which cross over orthogonally onto fan-like dendritic inputs to Purkinje cells. Granule cells provide context to sensorimotor information—indeed, the granule cells convey a topographically rich and fine-grained sensory map to the cerebellum necessary for fine motor control [115]. Purkinje cells are GABAergic neurons and are the only outputs from the cerebellar cortex. Mossy fibres carry sensory (from different sensory modalities such as the vestibular system) and efferent signals in different combinations to a massive number of granule cells. Mossy fibres convey low speed neural signals via the parallel fibres that intersect the Purkinje cells and impose time buffers of 50–100 ms. These delays require the predictions of the forward model. The Fujita model of the cerebellum comprises an adaptive filter that learns to compensate for time delays with parallel fibres forming delay lines but they are too short for this purpose [116]. Excitatory mossy fibres input an efference copy of motor commands to the forward model of the cerebellum. Climbing fibres from the inferior olive carry the motor error signals rather than input signals—each inhibitory Purkinje cell receives one climbing fibre but each climbing fibre may contact several Purkinje cells. Purkinje cell outputs from the cerebellar cortex project into the deep cerebellar nuclei which output to other parts of the brain. Outputs from the cerebellar nuclei project through the thalamus into the primary motor cortex (face, arm and leg), premotor cortex (arm), frontal eye field, and several areas of the prefrontal cortex [117]. In fact, those areas of the cerebral cortex that project to the cerebellum are also the targets of cerebellar outputs. The inverse dynamics model learning is located in the Purkinje cells to which the climbing fibre inputs carry error signals in motor torque coordinates thus acting as readout neurons. 

The cerebellum implements an associative learning algorithm involved in both motor and non-motor functions including higher cognition and language [118,119]. The cerebellum’s role in higher cognitive functions such as attention is less clear than its motor roles, but it performs the same computation on all its inputs [120,121,122]. The Marr–Albus–Ito cerebellar model emphasises motor learning aspects [123,124]—parallel fibre/Purkinje cell synaptic weights are modified by climbing fibre inputs from the inferior olive, i.e., learning input-output patterns. Long-term depression (LTD) alters synaptic connections onto Purkinje neurons during motor learning [125,126]. The cerebellum is a pattern recognition learning machine that learns predictive associative relationships between events (such as classical and operant conditioning) [127]. Associative learning involves extracting rules that predict the effects of stimuli with a given association weight. Classical conditioning involves a repeated presentation of a neutral conditioned stimulus (CS) with a reinforcing unconditioned stimulus (US). In classical conditioning, the CS (bell) is repeatedly paired with the US (food) to generate a CR (salivation) so that CR becomes the predictor of US. Similarly, eyeblink conditioning involves a tone (CS) and an airpuff to the eye (US) that generates a blink reflex [128]. The airpuff generates the eyeblink without learning (UR). The US (airpuff) is conveyed from the inferior olive by the climbing fibres while the CS (tone) is conveyed from the pontine nuclei by the mossy fibres. Both the pontine nucleus and inferior olivary nucleus (via the red nucleus) receive inputs from the cerebral cortex. The climbing fibres (US) “teach” the Purkinje cells to respond to parallel fibre inputs (CS). The animal learns to respond to the CS alone. After 100–200 training pairings, the tone is sufficient to induce the eyeblink response (CR that mimics UR). Temporal association is made only between the US and a preceding CS if the CR occurs prior to the US. The CS must precede the US by at least 100 ms with efficient conditioning occurring 150–500 ms before the US [129]. Surprising events (new evidence) imply increased uncertainty in prior beliefs about the world which incite animals to learn faster through classical conditioning, i.e., such learning is Bayesian [130]. In Bayesian analysis, the prior establishes expectations which if violated by observed evidence induces surprise. Hence, different prior models yield different rates of Bayesian learning. 

Inverse models are acquired through motor learning via Purkinje cell synaptic weight adjustment in the cerebellum. Motor learning involves the construction of an internal model representation in the cerebellum of the brain. Cause–effect relationships of each action must be learned inductively using training data generated by random movements [131]. Probabilistic forward models appear to influence random limb movements in infants (motor babbling) into specific motor spaces to reduce learning time [132]. Forward modelling involves training the transformation of joint torques to hand Cartesian coordinates onto a neural network. The forward model is a Bayesian network with learned probability distributions that also models prediction errors updated by observational feedback. This forward model can be used as a look-up table that computes the inverse model relating the joint torques given the hand coordinates. Furthermore, the forward model predicts the sensory outputs of motor inputs. The cerebellum implements paired forward and inverse models. Cerebellar-based motor deficiencies are some of the earliest indicators of autism prior to 3 years of age which appears to be due to over-reliance on proprioceptive feedback over visual feedback resulting in improper fine-tuning of internal models [133]. This favouring of internal stimuli over external stimuli manifests itself as stereotypical behaviour, social isolation and other cognitive impairments. The cerebellum appears to be a core hub that implements predictive capabilities across different cortical regions. The cerebellar cortex receives extensive inputs from the cerebral cortex but also sends its output to the dentate nucleus which projects widely to the different regions of the brain including to the prefrontal areas via the thalamus. The cerebellum receives inputs from the pontine nuclei that relay information from the cerebral cortex and outputs information back through the dentate nucleus and thalamus to the cerebral cortex. This cerebro-cerebellar loop may mediate tool use in humans [134].

It has been suggested that the cerebellum implements more complex forward models such as multitudes of Smith predictors [135]. The Smith predictor includes a forward model within an internal feedback loop to model a time delay. The latter delays a copy of the fast sensor predictions so that it can be compared directly with the actual sensory feedback to compensate for the time delay [136,137]. The forward model provides an estimate of the sensory effects of an input motor command which can be used within a negative feedback loop. The forward model acts as a predictive state estimator and a copy of the sensory estimate from the forward model is delayed to permit synchronisation and comparison with the actual feedback sensory effects of movement which are delayed by the feedback loop. Any errors between the predicted and actual sensory signal serve to improve performance and train the forward model. The linearised discrete system dynamics are given by:

(16) $$x_{i+1}=Ax_{i}+Bu_{i}$$

Time delays in the sensory feedback pathways require the use of predictive compensation such a Smith predictor [84]. The inner loop of the Smith predictor is based on the estimated state and the estimated dynamics:

(17) $${\hat{x}}_{i+1}=\hat{A}{\hat{x}}_{i}+\hat{B}{\hat{u}}_{i}$$

A predicted sensory estimate is used instead of the actual sensory measurement in a rapid internal high gain feedback loop to drive the system towards the desired sensory state. The linear control law is based on the difference between the desired state, estimated state, delayed state and estimated delayed state:

(18) $$u_{i}=K\left(x^{d}-\left({\hat{x}}_{i}-x_{d}-{\hat{x}}_{d}\right)\right)$$

Subtraction yields the error dynamics:

(19) $$e_{i}=x_{i+1}-{\hat{x}}_{i+1}=Ax_{i}+Bu_{i}-\left(\hat{A}{\hat{x}}_{i}+\hat{B}u_{i}\right)$$

If the forward model is perfect, $A=\hat{A}$ and $B=\hat{B}$:

(20) $$e_{i+1}=A\left(x_{i}-{\hat{x}}_{i}\right)=Ae_{i}$$

The Smith predictor also incorporates an outer loop with an explicit delay in the predicted sensory estimate to permit synchronisation with the actual sensory measurement—this is required to correct internal forward model errors. Hence, there are two forward models—one of the dynamics of the effector and the other of the delay in the afferent signal, the former with a faster learning rate of 100–200 ms and the latter with a slower learning rate of 200–300 ms [138]. This provides adaptability to the forward model through supervised learning. The Smith predictor utilises both a forward predictive model and a feedback delay model but the control loop time delay allows the delayed predictive model to be registered synchronously with actual sensory feedback to correct the estimated value through delay cancellation. The key to selecting which model the cerebellum performs is determined by the climbing fibre inputs—whether it carries predictive sensory information or motor command error information. An alternative approach to dealing with time delays is through wave variables. The cerebrospinal tract may represent control signals as wave variables of a standing wave formed by forward ($x_{m},{\dot{x}}_{m}$) and return ($x_{s},{\dot{x}}_{s}$) signals separated by a transmission delay T [139]. The cerebellum acts as a master wave variable that accepts u_s_(t-T) from the spinal cord and ${\dot{x}}_{m}$ from the cerebral cortex via the lateral reticular nucleus. These are combined with (x_m_-x_md_) to generate a reafferent wave variable ${\dot{x}}_{md}$ that ascends to the cerebral cortex and u_m_ that descends to the spinal cord. The signals (x_m_-x_md_) are generated through a reverberating interaction between the cerebellum and the magnocellular red nucleus by integrating $\left({\dot{x}}_{m}-{\dot{x}}_{md}\right)$. Feedback control is stable due to the passivity of the slave system. There are several theories of cerebellar function but all point towards the necessity for implementing predictive forward models.

## 10. Non-Cerebellar Motor Areas of the Brain

The basal ganglia receive inputs from all parts of the cortex associated with higher cognitive functions of attention, working memory, planning and problem-solving [140]. Indeed, it appears that the cerebellum, basal ganglia and cerebral cortex implement different types of learning in higher cognitive functions—supervised learning in the cerebellum from motor errors encoded in climbing fibres onto Purkinje cells, reinforcement learning in the basal ganglia based on reward signals encoded in dopaminergic fibres from the substantia nigra, and Hebbian unsupervised learning in the cerebral cortex for classification [141]. Dopamine neurons in the basal ganglia encode both current and predicted future rewards as the basis of reinforcement learning through the temporal difference algorithm. The basal ganglia have multiple inhibitory pathways. The striatum comprising the caudate nucleus and the putamen receives its main inputs from the cerebral cortex. It outputs to dopamine neurons and other neurons in the substantia nigra and to the globus pallidus. Outputs from the globus pallidus and substantia nigra are passed through the thalamus to the cerebral cortex. The dopamine neurons of the substantia nigra are associated with reward learning. The substantia nigra may also implement action selection. The cerebral cortex is involved in planning motor activity which feeds directly into the cerebellum and the basal ganglia for motor behaviour. It appears that both the cerebellum and basal ganglia project recurrently into the cerebral cortex and are involved in higher non-motor cognitive functions such as language and spatial processing. The basal ganglia project through the thalamus to the dorsolateral prefrontal cortex, lateral orbitofrontal cortex and the anterior cingulate cortex. The dentate nucleus of the cerebellum (massively expanded in hominids) projects through the thalamus to the dorsolateral prefrontal cortex (area 46 involved in spatial working memory) while it in turn projects into the globus pallidus of the basal ganglia forming a closed loop circuit [142]. The basal ganglia appear to be routing many output channels from the cerebral cortex through the thalamus back to the cerebral cortex. It is intimately connected with goal-directed behaviour.

## 11. The Centrality of Bayesian Inferencing

In the neocortex, feedforward connections follow from early processing areas to higher areas; feedback connections follow from higher areas to earlier processing areas. Lateral connections link areas within the same processing level. In the cortical column, outer layers implement predominantly (ascending) feedforward connections while inner layers implement predominantly (descending) feedback connections implying a predictive Bayesian inferencing process [143]. Feedforward connections convey prediction errors while feedback connections suppress prediction errors. The cerebral cortex has a uniform laminar architecture of six layers divided into specialised modalities with highly recurrent connections suggesting that the same fundamental algorithm may operate across the entire cortex. A candidate algorithm for the cerebral cortex is a Bayesian algorithm. Bayesian inference integrates top-down contextual priors implemented through feedback with bottom-up observations processed through the feedforward chain. The brain attempts to match bottom-up sensory data from the environment with top-down predictions by minimising the prediction errors (surprise) [144]. This Bayesian manifestation is equivalent to the free energy minimisation/maximum entropy model of expected energy minus the prediction entropy in the brain [145]. Free energy is minimised by choosing sensorimotor actions that reduce prediction errors (surprise)—this requires a Bayesian capability of updating predictive world models that cause the sensory data. The brain is indeed a Bayesian inferencing machine that estimates the contextual causes of its sensory inputs based on internal (forward) models which are essential for interpreting that sensory data [146,147]. The brain is a maximum a posteriori (MAP) estimator based on likelihoods modified by learned prior (forward) models that create expectations. Prior expectations are updated with new sensory evidence yielding a posterior estimate of the state of the world. The sensory data provides “grounding” to the prior model. Bayesian inferencing has been proposed as a model of memory retrieval, categorisation, causal inference and planning, i.e., Bayesian inferencing is implicated in all human cognition [148]. Bayesian inferencing is dependent on prior models of the world in memory which are upgraded with experience. The prior encapsulates information on the past history of inferences while the sensory data provides contextual framing. The tradeoff is between the predictability of future events (gain) against the cost of new hypothesis formation. Prediction defines the probability of an event given a set of features or cues—this constitutes the basis for defining causal laws with high probabilities (confidence) associated between the event and the cues, i.e., conditional learning.

The Bayesian approach provides the basis for probabilistic inductive inferencing. The joint probability of hypothesis h and observed data d is the product of the conditional probability of the hypothesis h given data d and the marginal probability of data d:

(21) P(h,d)=P(h|d)P(d)

We employ probabilities to represent degrees of belief in hypotheses *P*(*h*) prior to any observational data d—this is the prior probability which has a strong effect on the posterior probability. The posterior probability *P*(*h*|*d*) represents the probability of the hypothesis given the observational data d. Bayes theorem states that the revised (posterior) probability of an hypothesis is the product of its prior (former) probability and the probability of sensory data if the hypothesis were true (evidence). The likelihood and prior are combined and normalised using Bayes rule. Bayes rule relates the posterior probability of the hypothesis in terms of the prior probability:

(22) $$P\left(h|d\right)=\frac{P(d|h)P\left(h\right)}{P\left(d\right)}$$

where *P*(*d*|*h*) = probability of data given the hypothesis (likelihood). The prior smooths fluctuations in observed data. Likelihood weights the prior according to how well the hypothesis predicts the observed data. Maximum likelihood estimate (MLE) estimates parameters that maximise the probability of observed data *P*(*d*). Marginalisation is given in terms of competing hypotheses:

(23) $$P\left(h|d\right)=\frac{P(d|h)P\left(h\right)}{\sum_{h^{\prime }}P(d|h^{\prime })P\left(h^{\prime }\right)}$$

The likelihood is the probability (prediction) of sensory feedback based on the forward model given that context. There are multiple predictive forward models, each applicable in different contexts with a given likelihood in which the prior constitutes the probability of a context. 

Object perception may be regarded as a statistical visual inference process in which Bayesian techniques are employed to make optimal decisions based on prior knowledge and current noisy visual measurements [149]. The maximum a posteriori (MAP) estimate is proportional to the product of prior probability and the likelihood: p(S|I) = p(S,I)/p(I) where p(S,I) = p(I|S)p(S), S = scene description and I = image features. The Bayesian brain constructs complex models of the world despite sparse, noisy sensory data using a range of optimal filtering, estimation and control methods [150]. Bayesian probability enables inferencing with uncertain information by combining one or more sources of noisy sensory data with imperfect prior information. Bayesian estimation provides the vehicle for sensory fusion of multiple sensory modalities to enhance the quality of data for estimation. Predictive coding involves the elimination of redundancy by transmitting only unpredicted aspects of the sensory signal [151]. Top-down predictions filter out expected sensory inputs thereby reducing neuronal firing. This affords enhanced coding efficiency in processing sensory signals within the nervous system. This implicates prior predictive models as central to parsimonious information processing in the brain. The prediction error is processed to enhance unexpected (salient) stimuli. The predictive component represents hypotheses that explain sensory data while the prediction error that is transmitted further up the cortical hierarchy represents the mismatch between predicted and actual sensory data. Thus, the efficient minimum description length (MDL) neural code H=−logP(I|f)−logP(f) is equivalent to the Bayesian MAP estimation P(f|I)=P(I|f)P(f)/P(I) where *I* = input image, *f* = neural firing frequency representing visual features. The salient prediction error invokes better hypotheses (predictive models) further up the processing hierarchy—this requires that multiple hypotheses are available until the correct forward model has suppressed overall neural activity. The probability distributions involve the principle of least commitment but collapse to a single MAP estimate. In its simplest form, this is the basis of habituation in which repetitive stimuli invoke reduced neural response, i.e., conscious attention is not required. When the precision of noisy sensory data is low, attention is deployed to focus on ambiguous sensory data to increase its precision [152].

The brain combines visual information in retinal coordinates with proprioceptive information in head and body coordinates. The brain must compute coordinate transformations of Cartesian limb postures into limb joint movements in order to interact with objects in the environment. Coordinate transformations are performed in the posterior parietal cortex which represents multiple reference frames [149]. In Brodman’s area 7, reference frames are allocentric but in Brodman’s area 5, they are both eye- and hand-centred. In the lateral intraparietal layer, reference frames are both eye-centred and body-centred. These indicate multiple reference frames for coordinate transformations for relating visual stimuli in eye-centred frames to motor movements of the hand and arm in hand-centred frames through body-centred and head-centred reference frames. Sensorimotor transformations can be implemented through radial basis function neural networks using Gaussian function basis sets to associate sensory map coordinates with motor map coordinates [153]. This involves a one-to-many transformation as there are many sets of muscles that can implement a given Cartesian trajectory. A solution is to use two networks—an inverse model and a forward model which acts as a predictor.

A cerebral cortex neural network architecture has implemented Bayesian inferencing in the presence of noise [154]. It allows estimation of log posterior probabilities by quantitatively modelling the interaction between prior knowledge and sensory evidence. This Bayesian computation was implemented through a recurrent neural network with feedforward and feedback connections:

(24) $$y(t+1)=y(t)+\eta \left(-y(t)+W_{ff}I(t)+W_{fb}y(t)\right)$$

where *I* = network input, *y* = network output, *W_ff_* = feedforward weight matrix, *W_fb_* = feedback weight matrix, *η* = learning rate. The posterior probability of the hidden states of a hidden Markov model can also be computed through Bayes rule:

(25) $$\log p(x_{t}|y_{t},\ldots ,y_{1})=\log p(y_{t}|x_{t})+\log k+\log(p(x_{t}|x_{t-1})p(x_{t-1}|y_{t-1},\ldots ,y_{1})$$

where *x* = hidden states with transition probabilities *p*(*x_t_*|*x_t_*_-1_), y = observable output with emission probability *p*(*y_t_*|*x_t_*) and *k* = normalisation constant. These log likelihoods may be implemented on a recurrent neural network:

(26) $$y(t+1)=\log p(x_{t}|y_{t},\ldots ,y_{1})$$

(27) $$\eta W_{ff}I(t)=\log p(y_{t}|x_{t})$$

(28) $$\eta W_{fb}y(t)=\log\left(p(x_{t}|x_{t-1})p(x_{t-1}|y_{t-1},\ldots ,y_{1})\right)$$

The neural firing rates may be related to log posteriors computed by the neurons. The likelihood function is stored in the feedforward connections while the priors provide expected inputs encoded in feedback connections. Thus, knowledge of the statistics of the environment is stored in both sets of weights.

## 12. What Are Bayesian Networks?

To accommodate a large number of probability distributions, graphical Bayesian networks may be employed—they comprise nodes representing variables and directed edges associated with probability distributions representing causal dependencies between nodes. If a directed edge exists from node A to node B, A is the parent of child B. Every node is independent of other nodes except its descendants. Bayesian networks are acyclic so any node cannot be returned to itself via any combination of directed edges. Acyclicity ensures that the descendants of node *x_i_* are all reachable from *x_i_*. Singly connected Bayesian nets have any pair of nodes linked by only one path; multiply-connected Bayesian nets contain at least one pair of nodes connected by more than one path. A Bayesian network exhibits factorisation of the joint probability distribution within a directed acyclic graph [155]. Hence, the Bayesian network may be represented as:

(29) $$P\left(x_{1},x_{2},\ldots x_{n}\right)=\prod_{i}P(x_{i}|Pa\left(X_{i}\right))$$

where *Pa*(*X_i_*) = set of parents of *X_i_*. Bayesian networks represent the dependency structure of a set of random variables. A joint probability distribution on n binary variables requires 2^n^−1 branches. Dynamic Bayesian networks are Bayesian networks that represent temporal probability models by being partitioned into discrete temporal slices representing temporal states of the world. The edges in a Bayesian network define probabilistic dependencies but causal Bayesian networks assume that statistical correlation between variables does imply causation supported by probabilistic inferences [156]. The causal Bayesian network describes a causal model of the world in which parameter estimation determines the strength of causal relations. In this case, Bayes rule computes to what degree cause C has the effect of E. The degree of causation is given by:

(30) $$\Delta P=P\left(e^{+}|c^{+}\right)-P(e^{+}|c^{-})$$

where $P\left(e^{+}|c^{+}\right)$= conditional probability of the effect given the cause, $P(e^{+}|c^{-})$= conditional probability of the effect in the absence of the cause. An alternative measure is causal power (or weight) given by:

(31) $$W=\frac{\Delta P}{1-P(e^{+}|c^{-})}$$

These are both maximum likelihood estimates of causal strength relationships. Causal relations between multiple causes (including background causes) and a single effect may be implemented through the noisy-OR distribution. Children from the age of 2 can construct graphical causal (Bayesian) maps of the world based on causal relations between events [157]. Biological recurrent neural networks can implement Bayesian computations in the presence of noise. 

Predictiveness learned through frequency-based statistical probabilities is a stronger influence on decision-making than Bayesian (conditional) probability estimates [158]. Associative learning is based on the frequency of associations between predictive cue-outcome pairs, e.g., the Rescorla–Wagner rule which adjusts the association weight between cue-outcome pairs by minimising the prediction error [159]. The association weight establishes a causal relation between the cue and outcome according to the statistical coincidence between them. The Rescorla–Wagner learning rule iteratively computes the associative strength *W_i_* of a possible cause C with an effect E after trial n is given by:

(32) $$W_{i+1}=W_{i}+\Delta W_{i}$$

where $\Delta W_{i}=0$ if cause C does not occur;

= $\alpha \beta_{1}\left(\lambda -\sum^{\;}W_{i}\right)$ if both cause C and effect E occur;

= $\alpha \beta_{2}\left(0-\sum^{\;}W_{i}\right)$ if cause C occurs but not effect E.

In this way, causal relations are learned through experience that adjusts prior probabilities. Prior probability represents the structured background knowledge which is modified by new sensory information to enable inductive inferencing. A common model for the prior is the beta distribution given by:

(33) $$p\left(\theta \right)=\frac{\Gamma \left(\alpha +\beta \right)}{\Gamma \left(\alpha \right)\Gamma \left(\beta \right)}\theta^{\alpha -1}{\left(1-\theta \right)}^{\beta -1}$$

where $\Gamma \left(\alpha \right)=\int_{0}^{\infty }x^{\alpha -1}e^{-x}dx$ = gamma function, Γ(n)=(n−1)!. The parameters *θ*_i_ of the prior distribution may be learned by integrating out *α* and *β*:

(34) $$p\left(\theta_{i}|d_{1},d_{2},\ldots d_{n}\right)=\int^{\;}p\left(\theta_{i}|\alpha ,\beta ,d_{i}\right)p(\alpha ,\beta |d_{1},d_{2},\ldots d_{n})d\alpha d\beta$$

Exact Bayesian network inferencing is NP-hard so the integral is approximated using either Sequential Monte Carlo (such as recursive particle filters) or Markov Chain Monte Carlo methods.

Particle filters use a weighted set of samples to approximate probability density—they approximate the posterior probability distribution as a weighted sum of random samples from the prior distribution with weights given by the likelihood of measurement. Sequential Monte Carlo inferencing has been applied to selecting minimum prediction error solutions from multiple inverse-forward model pairs [160]. The posterior probability may be computed recursively:

(35) $$p\left(x_{i}|z_{1:i}\right)=\eta p(z_{i}|x_{i})\int^{\;}p\left(x_{i}|x_{i-1}\right)p\left(x_{i-1}|z_{1:i-1}\right)dx_{i-1}$$

where *p*(*x*_0_) = prior distribution, *p*(*z_i_*|*x_i_*) = observation model. Each particle represents a weighted hypothesis of an internal model, the weight $w_{i}^{k}$ being determined by the prediction error. The particles maintain an ensemble of hypotheses (principle of least commitment). This permits the detection of novelty when observations differ from predictions. The Kullback–Leibler divergence (relative entropy) between weighted particles and current observations defines the novelty. Markov Chain Monte Carlo methods determine the expected value of the function *f*(*x*) over a probability distribution *p*(*x*) of *k* random variables *x* = (*x*_1_,*x*_2_,…*x_k_*):

(36) $$\int^{\;}f\left(x\right)p\left(x\right)dx\approx \overset{m}{\underset{i=1}{\sum }}f\left(x_{i}\right)$$

In a Markov chain, the values of variables are dependent only on their immediate predecessor states and independent of previous states:

(37) $$p\left(x_{k}|x_{k-1},\ldots ,x_{1}\right)=p(x_{k}|x_{k-1})$$

Markov Chain Monte Carlo uses a Markov chain to select random samples according to a Markovian transition probability (kernel) function. As the length of the Markov chain increases, the transition kernel *K*(*x_i_*_+1_|*x_i_*) that gives the probability of moving from state *x_i_* to *x_i_*_+1_ converges to a stationary distribution *p*(*x*):

(38) $$p\left(x_{i+1}\right)=\underset{x}{\sum }p\left(x\right)K(x|x^{\prime })$$

The Gibbs sampler is a special case of the Metropolis–Hastings algorithm employed for successive sampling. A two-layer recurrent spiking network with a sensory layer of noisy inputs connected to a hidden layer can compute the posterior distribution *p*(*x_i_*|*z_i_*) among a population of neurons, i.e., Bayesian inferencing [161]. The forward weights encode the observation (emission) probabilities of sensory neurons. The recurrent weights from hidden neurons encode transition probabilities to capture the dynamics over time. Each spike in the posterior distribution represents an independent Monte Carlo sample (particle) of states with the variance of the spike count representing the mean value. Hebbian learning implements an expectation maximisation (EM) algorithm that maximises the log likelihood of hidden parameters given a set of prior observations. A neural particle filter model of the cerebellum exhibits a probability distribution of spiking which approximates a Bayesian posterior distribution in sensory data [162]. From this, it is possible to construct an optimal state estimate through Bayes rule to give the posterior distribution of spike measurement function in the cerebellum, $p\left(x|s\right)=\frac{p\left(s|x\right)p\left(x\right)}{p\left(s\right)}$ where *x* = environment state estimate, *s* = sensory spike measurement.

## 13. The Importance of Kalman Filters

The accommodation of noise in forward-inverse models and sensory feedback requires state estimation using observers. State estimation by an observer monitors an efference copy of motor commands and sensory feedback. The observer uses a recursive update process to estimate the state over time. The forward model is used to fuse sensory and motor information for predictive state estimation similarly to a Kalman filter model. Noisy sensory feedback combined with noisy forward models may be used to estimate the current state—an example is the Kalman filter that optimally estimates the current state by combining information from input signals and expectation-based signals [163]. The Kalman filter is an efficient version of the Wiener filter, with the latter using all previous data in batch mode while the former recursively uses immediately previous data. The Kalman filter is the optimal state estimator under conditions of Gaussian noise. The Kalman filter is a Bayesian method that computes the posterior probability of a situation based on the likelihood and the prior. It computes the relative weighting between the prior model and sensory data as the Kalman filter gain, a weighting that quantifies cognitive attention that balances prior predictive models and the likelihood of sensory data. The innovation (residual) term compares sensory input with the current prediction, i.e., a prediction error. The forward model acts as the predictor step while the sensory information provides the correction step. 

There are several approaches to sensor fusion—Bayesian probability, fuzzy sets and Dempster–Shafer evidence [164]—and the Kalman filter falls into the Bayesian approach. Dempster–Shafer theory is an extension of Bayes theorem to apportion beliefs and plausibility to hypotheses but is complex to compute. Fuzzy sets adopt partial set membership as a measure of imprecision to represent possibility rather than probability but it lacks the precision of Bayesian probability. In the Kalman filter model, the visual neural streams feed environmental information into an environment emulator (mental model) to generate expectations (predictions) for early visual processing to filter incoming data by directing attention and to fill in missing information. The superior colliculus integrates multisensory inputs in a manner consistent with Bayes rule to reduce uncertainty in orienting towards multisensory stimuli [165]. It receives inputs from visual, auditory and somatosensory brain systems and integrates them to initiate orienting responses such as saccadic eye movements. Neurons in the superior colliculus are arranged topographically according to their receptive fields. Shallow neurons of the superior colliculus tend to be unimodal while deep neurons are commonly multi-modal, e.g., visual-auditory. Deep neurons may compute the conditional probability that the source of a stimulus is present within its receptive field given its sensory input using Bayes rule that reduces the ambiguity from individual senses alone. For a single (e.g., visual) input alone, the posterior probability of a target *t* given a visual stimulus *s* in the receptive field is given by:

(39) $$p(t|v)=\frac{p(v|t)p(t)}{p(v)}$$

where *p*(*v*|*t*) = likelihood of a visual stimulus given the target (characteristic of the vision system) modelled as a Poisson distribution of neural impulses, $p(v)=\frac{\lambda^{f}e^{\geq \lambda }}{f!}$= probability of a visual stimulus regardless of a target (characteristic of the vision system), *p*(*t*) = prior probability of the existence of the target (property of the environment), *f* = number of input neural impulses per 250 ms, λ = mean number of spontaneous neural impulses. The bimodal case (e.g., visual and auditory inputs) is computed similarly:

(40) $$p(t|v,a)=\frac{p(v,a|t)p(t)}{p(v,a)}$$

It is assumed that the sensory modalities are conditionally independent, so *p*(*v*.*a*) may be computed as a product of the corresponding individual likelihoods: p(v,a)=p(v)p(a) and p(v,a|t)=p(v|t)p(a|t). The multisensory case follows similarly.

In the brain, the integration of multisensory data (such as visual and vestibular data) occurs in the medial superior temporal (MST) area in which population coding implements sensory cue weighting [166]. The Bayesian estimator computes the posterior probability distribution of state *x_i_* at time *i* given the prior distribution *p*(*x_i_*|*z_i_*_-1_), likelihood function *p*(*z_i_*|*x_i_*) and history of measurements *z*_1_,…,*z_i_*:

(41) $$p\left(x_{i}|z_{i}\right)=\frac{p\left(z_{i}|x_{i}\right)p(x_{i}|z_{i-1})}{p(z_{i}|z_{i-1})}$$

**Figure 4:**
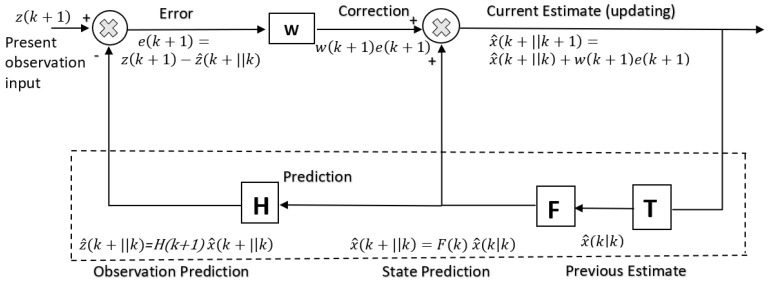
Kalman filter computational process.

This is recursive so new measurements constantly update the posterior probability of the state. The Kalman filter assumes a linear Bayesian system corrupted by Gaussian noise. The Kalman filter estimates the state *x*(*t*) of the system (notationally, caps indicate estimates) (Figure 4). 

The model prediction step is given by a prior model $\hat{x}(t|t-1)$:

(42) $$\hat{x}(t|t-1)=A\hat{x}(t-1|t-1)+w\left(t\right)$$

(43) $$P(t|t-1)=\mathrm{AP}(t-1|t-1)A_{}^{T}+Q\left(t-1\right)$$

where Q = cov(w(t)) of process noise, P = error covariance. The measurement update step is given by the posterior estimate $\hat{x}(t|t)$ based on the prediction error $z\left(t\right)-H\;\hat{x}(t|t-1)$:

(44) $$\hat{z}(t|t-1)=H\;\hat{x}(t|t-1)+v\left(t\right)$$

(45) $$S\left(t\right)=\mathrm{HP}(t|t-1)H_{}^{T}+R\left(t\right)$$

(46) $$K\left(t\right)=P(t|t-1)H_{}^{T}S{\left(t\right)}_{}^{-1}=\mathrm{Kalman}\;\mathrm{filter}\;\mathrm{gain}$$

(47) $$\hat{x}(t|t)=\hat{x}(t|t-1)+K\left(t\right)(z\left(t\right)-H\;\hat{x}(t|t-1))$$

(48) P(t|t)=(I−K(t)H)P(t|t−1)

where R = cov(v(t)) of measurement noise. Hence, the predicted state $\hat{x}(t|t)$ is given by the process model estimate $\hat{x}(t|t-1)$ subject to a correction of the Kalman gain K(t) applied to the prediction error $\left(z\left(t\right)-H\;\hat{x}(t|t-1)\right)$ with an uncertainty in the estimate given by the covariance P(t|t). The Kalman filter has a diversity of uses. The Kalman filter may be exploited as an adaptive observer for parameter estimation for system identification in a control system—model reference adaptive control has been exploited to perform such parameter adaptation [167]. The active observer algorithm reformulates the Kalman filter as a model reference adaptive control system [168]. Model predictive control may be robustified using a Kalman filter as a state estimator based on minimum mean square error to yield enhanced performance [169,170]. A neural network may be represented as a state estimation process with a system state model $w_{k+1}=w_{k}+v_{pk}$ and a measurement model $y_{k}=f_{k}\left(w_{k}x_{k}\right)+v_{mk}$ where $v_{pk}$= process noise, $v_{mk}$= measurement noise, $f_{k}\left(.\right)\;$= neural network. The Kalman filter is a stochastic version of the Luenberger observer with an optimal gain which fits the notion of noisy neurons. A Kalman filter estimator may be employed to estimate the weights of a neural network based on the residual $z^{d}\left(t\right)-z\left(t\right)$ between desired and actual outputs:

(49) $$w\left(t+1\right)=w\left(t\right)+K\left(t\right)\left[z^{d}\left(t\right)-z\left(t\right)\right]$$

where $z\left(t\right)=H^{T}w\left(t\right)$. A neural network has implemented a PID controller in which the learning rule is given in terms of Kalman filter parameters [171]:

(50) $$w\left(t+1\right)=w\left(t\right)-\eta \frac{dJ\left(t\right)}{dw\left(t\right)}$$

where $J\left(t\right)=\left(1-\lambda \right)\left(H^{T}\left(t\right)P\left(t\right)H\left(t\right)+R\right)+\left(H^{T}\left(t\right)x\left(t\right)-z\left(t\right)\right)$

The backpropagation algorithm employed by the multilayer perceptron (MLP) neural network may be represented in Kalman filter form [172]:

(51) $$\Delta w_{k}\left(t\right)=\eta \overset{n}{\underset{k=1}{\sum }}\left(z_{k}^{d}-z_{k}\right)\frac{\partial z_{k}}{\partial w\left(t\right)}$$

The learning rate *η* may be equated to extended Kalman filter parameters:

(52) $$\eta =S{\left(t\right)}^{-1}P\left(t\right)$$

The backpropagation (gradient descent) algorithm does not update the P matrix but the Kalman filter form decreases the weight update rate as training proceeds. The filtered output is a weighted combination of past input signal samples. Hence, the backpropagation algorithm learns weights of the MLP in batch mode with an arbitrary learning rate and is a degenerate form of the extended Kalman filter that learns weights incrementally with an optimally adjusted learning rate [173]. The Kalman filter approach for training the weights of a multilayer perceptron is superior to the backpropagation algorithm in that it requires fewer iterations for convergence at the cost of three orders of magnitude greater computational complexity [174]. Kalman filters may offer a means to construct a world model which allows prediction of future events from prior experience by combining multiple stimuli through predictive networks [175]. The Kalman filter has been used to enhance the genetic algorithm (GA) in the Kalman-extended GA (KGA) [176]. In this case, the Kalman-estimated fitness value of solutions are uncertain due to nonstationary environments (process noise with variance Q) and due to corrupted evaluations (measurement noise with variance R). Each fitness estimate has an initial associated uncertainty P = P_prior_+Q. This yields an estimated posterior fitness value of $f=f_{\mathrm{prior}}+\frac{P_{\mathrm{prior}}}{P_{\mathrm{prior}}+R}\left(g-f_{\mathrm{prior}}\right)$ and an updated posterior uncertainty $P=\frac{P_{\mathrm{prior}}R}{P_{\mathrm{prior}}+R}$. KGA selects the individual with the lowest uncertainty with fitness greater than average.

The Kalman filter is a state predictor for linear systems with Gaussian noise but the extended and unscented Kalman filters extend its validity to nonlinear systems with non-Gaussian noise. The unscented Kalman filter (UKF) can approximate the a posteriori probability density of the state as a Gaussian distribution with a mean and covariance to 3rd order accuracy. The UKF approximates the state statistics with a Gaussian random variable represented by a set of weighted sample points. These sigma points are propagated through the nonlinear system generating posterior sigma points which converge to the true mean and covariance. The UKF procedure begins with initialisation of the prior state $x_{0}=<x_{0}>$ and the prior covariance $P_{0}=<(x_{0}-{\hat{x}}_{0}){\left(x_{0}-{\hat{x}}_{0}\right)}^{T}>$. Sigma points are selected based on the state estimate:

(53) $$\chi_{j.t-1}^{i}={\hat{x}}_{i}\left(t-1\right)\;\mathrm{for}\;j=0$$

(54) $$\chi_{j.t-1}^{i}={\hat{x}}_{i}\left(t-1\right)+{\left(a\sqrt{{\mathrm{nP}}_{i}\left(t-1\right)}\right)}_{j}\;\mathrm{for}\;j=1,2,\ldots ,n$$

(55) $$\chi_{j.t-1}^{i}={\hat{x}}_{i}\left(t-1\right)-{\left(a\sqrt{{\mathrm{nP}}_{i}\left(t-1\right)}\right)}_{j-n}\;\mathrm{for}\;j=n+1,\;n+2,\ldots ,2n$$

where a = tuning parameter that determines the spread of sigma points around ${\hat{x}}_{i}\left(t-1\right)$. The predicted state mean and covariance are given by:

(56) $${\hat{x}}_{i}\left(t|t-1\right)=\overset{2n}{\underset{j=0}{\sum }}w_{j}\chi_{j,t|t-1}^{i}$$

(57) $$P_{i}\left(t|t-1\right)=\overset{2n}{\underset{j=0}{\sum }}w_{j}\left(\chi_{j,t|t-1}^{i}-{\hat{x}}_{i}(t|t-1)\right){\left(\chi_{j,t|t-1}^{i}-{\hat{x}}_{i}(t|t-1)\right)}^{T}+Q$$

where $w_{j}=1-\frac{1}{a^{2}}$ for j = 0.

$w_{j}=1-\frac{1}{2{\mathrm{na}}^{2}}$ for j = 1,2,…,2n.

$\chi_{j,t|t-1}^{i}=f\left(\chi_{j,t|t-1}^{i}\right)$ for j = 0,1,…,2n.

A new set of sigma points with new mean and covariances are selected:

(58) $$\chi_{j,t|t-1}^{i}={\hat{x}}_{i}(t|t-1)\;\mathrm{for}\;j=0$$

(59) $$\chi_{j,t|t-1}^{i}={\hat{x}}_{i}\left(t|t-1\right)+{\left(a\sqrt{{\mathrm{nP}}_{i}(t|t-1)}\right)}_{j}\;\mathrm{for}\;j=1,2,\ldots ,n$$

(60) $$\chi_{j.t|t-1}^{i}={\hat{x}}_{i}\left(t|t-1\right)-{\left(a\sqrt{{\mathrm{nP}}_{i}(t|t-1)}\right)}_{j-n}\;\mathrm{for}\;j=n+1,\;n+2,\ldots ,2n$$

The weighted mean and covariance of the predicted measurement are given by:

(61) $${\hat{z}}_{i}\left(t|t-1\right)=\overset{2n}{\underset{j=0}{\sum }}w_{j}\gamma_{j,t|t-1}^{i}=\overset{2n}{\underset{j=0}{\sum }}w_{j}h\left({\chi^{\prime }}_{j,t|t-1}^{i}\right)$$

(62) $$P_{i}\left({\hat{z}}_{i}(t|t-1\right))=\overset{2n}{\underset{j=0}{\sum }}w_{j}\left(\gamma_{j,t|t-1}^{i}-{\hat{z}}_{i}(t|t-1)\right){\left(\gamma_{j,t|t-1}^{i}-{\hat{z}}_{i}(t|t-1)\right)}^{T}+R$$

where $\gamma_{j,t|t-1}^{i}=h\left(\chi_{j,t|t-1}^{i}\right)$ for j = 0,1,…,2n. The new state estimate and error covariance are updated as:

(63) $$P_{i}\left({\hat{x}}_{i}(t|t-1\right)\left({\hat{z}}_{i}(t|t-1\right))=\overset{2n}{\underset{j=0}{\sum }}w_{j}\left(\chi_{j,t|t-1}^{i}-{\hat{x}}_{i}(t|t-1)\right){\left(\gamma_{j,t|t-1}^{i}-{\hat{z}}_{i}(t|t-1)\right)}^{T}$$

(64) $$K_{i}\left(t\right)=P_{i}({\hat{x}}_{i}(t|t-1){\hat{z}}_{i}(t|t-1)P_{i}^{-1}\left({\hat{z}}_{i}\left(t|t-1\right)\right)$$

(65) $${\hat{x}}_{i}\left(t\right)={\hat{x}}_{i}\left(t|t-1\right)+K_{i}\left(t\right)\left[z_{i}\left(t\right)-{\hat{z}}_{i}\left(t|t-1\right)\right]$$

(66) $$P_{i}\left(t\right)=P_{i}\left(t|t-1\right)-K_{i}\left(t\right)P_{i}\left({\hat{z}}_{i}\left(t|t-1\right)\right)K_{i}^{T}\left(t\right)$$

The UKF may be rendered adaptive to prior model errors through an MIT rule-based approach that updates the elements of the covariance matrix Q of process noise [177]:

(67) $$q_{i}=q_{i-1}-\eta \frac{\partial V_{i}}{\partial q_{i-1}}T$$

where $V_{i}=tr{\left(S_{i}-{\hat{S}}_{i}\right)}^{2}$, $S_{k}=\frac{1}{n}\sum_{k=i-n}^{i-1}v_{k}v_{k}^{T}$. An attractor-based recurrent neural network has been shown to implement a one-dimensional Kalman filter under conditions of small prediction error [*z*(*t*+1)-$\bar{x}$(*t*+1)] [178]:

(68) $$\hat{x}\left(t+1\right)\approx \bar{x}\left(t\right)+\frac{A\left(t+1\right)}{\alpha \left(t+1\right)}\left[z\left(t+1\right)-x\left(t+1\right)\right]$$

where $A\left(t\right)\propto \frac{1}{\sigma_{x}^{2}}$, $\alpha \left(t\right)\propto \frac{1}{{\hat{\sigma }}_{x}^{2}}$. It has been shown that the UKF outperforms the standard Kalman filter and Wiener filter in the task of inferring hand motion from populations of primary motor cortex neuron firing [179].

It may be assumed that hand kinematics x are a function of neural firing z with x = f_1_(z) while neural coding assumes that neural activity is a function of hand kinematics with z = f_2_(x)+n. Hand motion $x_{i}={\left(x,y,v_{x},v_{y},a_{x},a_{y}\right)}^{T}$ can be inferred from the firing rates of populations of neurons in the primary motor cortex M1 based on Bayesian methods [180]. Bayesian inference involves calculating the posterior probability of hand movement based on measurements of a sequence of neural firing rates. The likelihood models the probability of firing rates given a specific cartesian hand movement ($z_{i}=H_{i}x+v_{i}$ where *H_i_* = measurement matrix that relates hand state to neural firing rates) while the prior constitutes a probabilistic model of hand kinematics ($x_{i+1}=A_{i}x_{i}+w_{i}$ where *A_i_* = state matrix describing the evolution of the hand state with time). The Kalman filter performs population decoding of the motor cortex to extract cartesian hand movement from a population of cortical neurons to control a 2D cursor:

(69) $$p\left(z_{i}|x_{i}\right)=\overset{M}{\underset{k=1}{\prod }}p(z_{i,k}|x_{i})=\overset{M}{\underset{k=1}{\prod }}\frac{1}{\sqrt{2\pi }v_{i}}exp\left(-\frac{1}{2}{\left(z_{i,k}-H_{k}x_{i}\right)}^{2}/v_{i}^{2}\right)$$

A recurrent neural network implementation of the Kalman filter can construct internal models for predicting sensory consequences of motor action while integrating sensory data in tasks such as locating objects while moving our eyes and driving our arm to acquire an object located in the world [181]. The recurrent neural network comprises a single layer of sensory and motor input neurons with recurrent connections that predict future sensorimotor states given the current sensory input and efferent motor outputs. Population codes encode (symbolic) variables as patterns of activity in groups of neurons, each with overlapping Gaussian-shaped tuning curves. The internal model computed from the initial state without sensory feedback accumulates motor errors so sensory feedback is essential. Sensorimotor data is noisy and the shape of the tuning curves determine the noise covariance. The sensorimotor neurons form a topographic map. The maximum a priori estimate is given by the Kalman filter: $\hat{x}\left(t+1\right)=\left(I-K\left(t\right)\right)\left[A\hat{x}\left(t\right)+Bu\left(t\right)\right]+K\left(t\right)\hat{x}\left(t\right)$ where *x*(*t*) = sensory outputs, $K\left(t\right)=\Sigma \left(t\right)\Sigma {\left(t\right)}^{-1}$= Kalman gain, Σ(*t*) = covariance of Kalman filter estimates, Σ(0) = covariance of initial state (prior), *u*(*t*) = input motor commands. The prediction generated by the internal model is combined with proprioceptive feedback from muscle sensors by minimising the mean square error of the estimate. 

The brain is incapable of manipulating vectors and matrices in Kalman filter computations for state estimation but probability distributions are represented through firing rates f among a population of neurons—this comprises a probabilistic population code, i.e., information about a variable is represented by a pattern of neural activity over a population of neurons representing the likelihood of the stimulus s, p(f|s). For example, the direction of arm movement while reaching is coded by neurons that fire at a rate that varies with the cosine of the angle from the neuron’s maximum sensitivity to the direction of movement. These cosine tuning curves are characterised by a maximum and a standard deviation. A recurrent exponential family harmonium (EFH) neural network can learn from probabilistic population code inputs and estimate the states of a linear dynamical system without supervision [182]. The EFH is a generalisation of the restricted two-layer Boltzmann machine with full interlayer connections but no intralayer connections. The inputs presented to the input layer are proprioception and downstream layer responses from the previous timestep. The recurrent feedback connections permit learning of the dynamic properties of the stimuli. The number of recurrent neurons is the same as the number of downstream (hidden) neurons. Learning of the sensory weights from the sensory to hidden layers w_s_ and the recurrent weights from the recurrent to hidden layers w_r_ is Hebbian, enabling tracking of moving stimuli locations including own limb locations. The recurrent loop emulates reciprocal connections in the parietal cortex that generates characteristic rhythmic activity. A biologically inspired neural network controller model has been proposed for the feedforward ballistic movements of the human arm which replicates the straight-line position profiles and bell-shaped velocity profiles of the hand [183]. Ballistic movements rely on feedforward models as feedback from visual or proprioceptive sensors introduces time delays.

In hierarchical visual processing, feedback connections from higher to lower level visual cortical areas convey expectations (predictions) while forward connections transmit residual errors between low level visual inputs and the predicted estimates [184]. The feedforward channel generates hypotheses while feedback from higher visual areas imposes prior models that influence the earlier levels. Estimated predictions of responses at the next lower level through feedback connections are used at all stages of visual processing. Similarly, the error between predicted and actual responses are transmitted to the next higher level through feedforward connections. This includes the centre-surround responses of retinal and lateral geniculate nucleus neurons, i.e., only regions of the visual field with high contrast both spatially (retina) and temporally (lateral geniculate nucleus) are processed. The larger receptive fields of higher neurons integrate information from larger spatial fields to influence lower neurons with smaller receptive fields. This is enabled through reciprocal connections between cortical areas for feedback of predictions to earlier processing and the convergence of feedforward processing with larger receptive fields to pass residuals between data-driven and expectation-driven processing. Hence, cortico-cortical connections in the hierarchical visual cortex are reciprocal. Hierarchical Bayesian inference integrates top-down contextual priors that are predictive and implemented through feedback with bottom-up posterior observations implemented through the feedforward chain to estimate the current state in an expectation-maximisation algorithm [185]. Bayes theorem allows hidden variables *x_h_* to be inferred from contextual variables *x* and observations *y*:

(70) $$p(y,x|x_{h})=p(y|x,x_{h})p(x|x_{h})$$

Now, $p(x|y,x_{h})p(y|x_{h})=p(y,x|x_{h})$ so the posterior probability is given by:

(71) $$p(x|y,x_{h})=\frac{p(y|x,x_{h})p(x|x_{h})}{p(y|x_{h})}$$

where *p*(*x*|*x_h_*) = prior probability. Cortical areas V1 and V2 determine the most probable value of *x* by finding the posterior probability that maximizes *p*(*x*|*y*,*x_h_*), i.e., it multiplies forward propagating *p*(*y*|*x*) with feedback *p*(*x*|*x_h_*). Further processing in different visual areas forms a Markov chain. The top-down prior re-shapes the posterior probability distribution by focussing attention. A particle filter maintains a weighted set of posterior probabilities (multiple hypotheses) through a forward/backward algorithm to propagate beliefs about the state of the world. This explains the phenomenon of binocular rivalry. 

An extended Kalman filter may implement this hierarchical visual process. It optimally estimates the current state of visual recognition I(t)=w(t)x(t)+n(t) by combining information from input signals and expectation-based predicted signals where $e\left(t\right)=\hat{x}\left(t\right)-x\left(t\right)$ [186]. The prediction process compensates for signal propagation time delays. The feedback synaptic weights are adapted using Hebbian learning gradient descent to minimise the prediction error $w\left(t+1\right)=w\left(t\right)+\eta \left(I-w\left(t\right)x\right)x^{T}$ where *I* = input vector, *η* = learning rate, *x* = state vector (presynaptic activity), (*I*-*wx*) = postsynaptic activity. A weight decay component may be added. The feedforward weight matrix should be related to the feedback weight matrix by $W_{ff}=W_{fb}^{T}$. The forward pathway carries residuals between the current state *x* and the feedback predicted state *x_d_* according to an extended Kalman filter which attempts to minimize the reconstruction error $x_{d}(t)=x(t)+n(t)$ where *n*(*t*) = noise with a noise covariance matrix. The Kalman filter implements an expectation maximisation algorithm to compute the optimal state estimate as $x\left(t\right)=\hat{x}\left(t\right)+Pw^{T}\Sigma_{bu}^{-1}\left(I-w\hat{x}\left(t\right)\right)+P\Sigma_{td}^{-1}\left(\hat{x}\left(t\right)-x\left(t\right)\right)$ where Σ = noise covariance matrices (bottom-up and top-down). The estimate weights the bottom-up and top-down contributions according to their respective noise covariances and the innovations. Similarly, the neural particle filter is a sampling-based nonlinear Bayesian inferencing filter implemented in a neural network [187]. 

It has been suggested that the cerebellum acts as a Kalman filter state estimator for motor trajectory prediction [188]. In this model, the Kalman filter estimates the state (Cartesian position/velocity) of the hand using both motor output and sensory feedback in conjunction with a model of the motor system. There are two sources of uncertainty—the motor response (model) errors and sensory feedback (measurement) errors. The discrete dynamic state model is given by:

(72) $$x_{k+1}=Ax_{k}+w_{k}$$

where *w_k_* = process noise with covariance Q_k_. The measurement model is given by:

(73) $$y_{k}=Cx_{k}+v_{k}$$

where *v_k_* = measurement noise with covariance *R_k_*. The optimal state estimate computed by secondary neurons in the vestibular nuclei is given by:

(74) $${\hat{x}}_{k+1}=[A-K_{k}C]{\hat{x}}_{k}+K_{k}y_{k}$$

where $K_{k}=APC^{T}{\left[CPC^{T}+R_{k}\right]}^{-1}$ = Kalman gain. The Kalman filter gain determines the coupling strength between measurement data and the current state estimate. This adjusts the Purkinje connection weights between primary and secondary vestibular neuron layers. Covariance of the current state estimate is given by the matrix Riccati equation:

(75) $$P_{k}=AP_{k-1}A^{T}+Q_{k-1}-AP_{k-1}C^{T}{\left[CPC^{T}+R_{k-1}\right]}^{-1}{\left[AP_{k-1}C^{T}\right]}^{T}$$

The Kalman filter combines the feedforward model with sensory feedback: the feedforward model predicts the motor response between states while the feedback compares predicted sensory effects with the current sensory values. The Kalman filter tends to be over-confident in its model so the climbing fibres act as a teacher signal to provide error corrective feedback. The sensory error is used to correct the state estimate from the forward model. The forward model must be adaptable to feedback errors. The form of these internal models may vary between two extremes from an input-output lookup table which has high storage costs to a parametric model which has high processing costs. Discrepancy between the estimated forward model sensory outputs and actual sensory outputs must be used to update the model as a function approximator using supervised learning methods. An optimal representation is through basis functions with restricted receptive fields to represent mappings by combining basis functions.

It has been suggested that the cerebellum is a weighted adaptive filter and that the Smith predictor and Kalman filter are biologically implausible [189]. However, the chief criticism has been in how modular processes can be partitioned in the brain similarly to their partitioning in logical algorithms. It is well known that neural partitioning can be substantially different from logical partitioning [190] so this does not constitute an effective argument against such algorithms being implemented neurally. The Kalman filter is highly computationally efficient and effective. Recent consensus favours a general forward model of the cerebellum over the Smith predictor and the Kalman filter [191]. However, the Kalman filter model successfully predicts that relative weightings shift from the forward model at the start of movement to sensory feedback through subsequent movement reproducing the properties of human trajectory movements [192]. It has nevertheless been questioned whether the Kalman filter as a Bayesian form of the Rescorla–Wagner learning rule (encoded as $\eta ={\left(R+H^{T}PH\right)}^{-1}$) is employed in associative learning of backward blocking though other Bayesian formulations may be consistent [193]. The question is still open.

State prediction using the forward model reduces the effects on sensory feedback delays. Sensory prediction can be derived from the state prediction which may be used to cancel self-generated sensory signals (such as suppression of tickle stimulation if self-applied). State estimation and prediction in the brain appears to be performed by the parietal cortex [194]. The posterior parietal cortex is involved in skilled motor activity which requires locating of visual targets using egocentric eye movements—it computes motor errors to permit visually-directed motor correction [195]. To compute this error, it requires the current target position in body-centred coordinates and the current position of the end effector by transforming proprioceptive data from the somatosensory cortex and efferent joint torque commands from the motor cortex into end effector coordinates. The posterior parietal cortex merges arm-referenced stimuli (such as proprioception and efferent copies) and target-referenced stimuli (such as vision) into a common reference frame required for generating motor errors. Lesions in the right parietal cortex yields the body neglect phenomenon where body parts on one side are regarded as alien—this is consistent with the Kalman filter model. The emulation theory of representation posits that the brain actively constructs forward models (emulators) of the body and its environment during motor learning [196]. The emulator institutes the a priori dynamic model and the measurement estimate of a Kalman filter while actual measurements are fed back to generate the residual. The Kalman filter model allows the use of initial state estimates based on process model predictions which only later are corrected with proprioceptive feedback. A low Kalman gain which favours the model prediction evolves into a higher Kalman gain which favours emphasis on sensory information.

## 14. Imitation Learning is Facilitated by Forward Models

Visual imagery activates the primary visual cortex and the premotor cortex in an off-line mode providing simulated sensory inputs to an emulator model. Actual and imagined movement activates the same premotor and supplementary motor areas of the brain. During imagery, the Kalman gain filters out sensory information to exploit the emulator dynamic model [196]. Furthermore, motor commands are inhibited although efferent copies are sent to the emulator for simulation. The forward model permits mental rehearsal of motor movements by performing motor movements while disabling signals propagated to the muscles. During motor imagery, the primary motor cortex is inactive while the supplementary motor areas, premotor cortex and the cerebellum are active. The basal ganglia have traditionally been associated with action selection including inhibition of actions during planning. This provides the basis for predicting future states generated by simulating but not performing actions. An example of such simulations is imagining mental movement of objects [197]. 

Specific neurons—mirror neurons—in the premotor areas are activated if an individual performs an action or observes another individual performing an action [198]. Mirror neurons in area F5 of the macaque frontal/premotor cortex are responsible for motor representation—they respond when a primate performs a particular self-generated motor action and when observing another primate perform a similar action. Mirror neurons provide the basis for understanding motor actions and their intention. The STS-PF-F5 pathway provides the basis for matching between the visual representations in superior temporal sulcus (STS), the organisation of goal-oriented actions in the prefrontal (PF) cortex and motor primitives represented in F5. There are reciprocal connections between these regions that are learned during imitation. Learning through imitation requires certain capabilities: (i) mapping visual and proprioceptive sensory inputs into motor outputs; (ii) compensating for morphological differences between demonstrator and imitator; (iii) inferencing of the demonstrator’s intention by the imitator from observations of the demonstrator’s motor actions. This requires the capture of intent as well as motor skills in order to determine the sequence of subgoals subject to environmental variations, noise and imprecision. Robotic learning by imitation of sequential grasping and manipulation involves two processes [199]: (i) imitation through replication of the results of observed actions rather than replication of the actions themselves; (ii) understanding of the intentions of the observed agent based on motor simulations. Robots can learn and imitate movements of a human being by combining motor schema primitives which are activated similarly to mirror neurons [200]. The correspondence problem involves converting perceptual observations into its own motor responses—this requires the existence of predictive forward models. Representing motor primitives as predictive forward models allows observations to be matched to the motor primitives in the same agent-based coordinate system. Motor primitives in F5 must be translated into postures through kinematic transformations to convert desired hand Cartesian coordinates into joint coordinates. This may be realised through a recurrent neural network model storing a number N of configurations $\theta_{j}=\left(\theta_{j1},\ldots ,\theta_{jn}\right)$ for *j* = 1,…,N to map the Cartesian workspace. Topographically, each neuron represents a single posture *θ_j_* which is connected to its k nearest neighbours defined by a Euclidean distance metric $d_{ik}=\left|\theta_{i}-\theta_{k}\right|$ and bidirectional connection weight $w_{ik}=\exp\left(d_{ik}\right)$ assuming a Hopfield net configuration. The Cartesian goal posture input to the network generates a spreading activation wave which reaches the neuron corresponding to the initial position. A sequence of postures from the initial to goal states is defined by a backpropagated path through the spreading activation wave. It is not clear how far motor intention was captured in this model. A hierarchy of neural net models has been developed which emulate the spinal cord, primary motor cortex, premotor cortex, cerebellum and temporal cortex for visuo-motor control a robotic manipulator through imitation [201].

Internal forward models may provide the basis for predicting the sensory consequences of imitated actions. This could be the key to imitation in determining intentions from motor actions. The internal body model may be extended to incorporate external objects like tools. An internal world model is an expansion of the internal body model embedded within the external environment. Internal world models are essential to explain the 500 ms time delay in subjective sensations being backprojected to coincide with the onset of stimulation. The internal models employed to generate goal-directed actions may be employed to simulate the motor actions of others and infer their intentions from prior goal knowledge. The predictor–controller system may be adapted to imitation learning [202]—sensory predictions are compared with the demonstrator’s observed sensory state. If there are deviations, the agent’s model of the other agent may be refined, i.e., a social model that may be employed to access hidden mental states in others. In this case, our own body serves as the model to predict the behaviour of others because the mental states of others are inaccessible. Learning by imitation is a Bayesian method that uses observations and internal sensorimotor models [203]. Imitation learning involves estimating hidden states from a sequence of observations—this may be implemented through the forward-backward algorithm for hidden Markov models. The sequence of states constitutes a sequence of subgoals to reach an overall goal state. We select action a_i_ that maximises the probability of transitioning from state *s_i_* to *s_i_*_+1_ towards goal *s_j_*, *p*(*a_i_*|*s_i_*,*s_i_*_+1_,*g* = *s_j_*). This constitutes the inverse model. The forward model *p*(*s_i_*_+1_|*s_i_*,*a_i_*) is independent of the goal state and the prior *p*(*a_i_*|*s_i_*,*g* = *s_j_*) are both learned through motor learning. The forward model predicts the sensory consequences of actions. Bayes theorem applied to the forward model and prior gives the inverse model:

(76) $$p\left(a_{i}|s_{i},s_{i+1},g=s_{j}\right)=\frac{p\left(s_{i+1}|s_{i},a_{i}\right)p(a_{i}|s_{i},g=s_{j})}{p(s_{i+1}|s_{i},g=s_{j})}$$

The MAP algorithm selects actions *a_i_* that maximise this value. Imitation must also establish a mapping between observed object-oriented motor actions of another agent into egocentric self-motor actions. The forward and inverse models may be used to infer the intent of the observed agent by application of Bayes rule, *p*(*g* = *s_j_*|*a_i_*,*s_i_*,*s_i_*_+1_). Imitation learning in humans is a biological phenomenon that has leveraged human culture in diverse habitats and surviving in very different physical environments throughout the world by adopting different cultural adaptations [204]. Bayesian estimation of terrain costs according to detected features vector have been exploited in imitation learning of robot navigation [205]. Imitation learning in conjunction with reinforcement learning has been applied to the problem of robot grasping using dynamic motion primitives [206]. Learning by observation [207] and programming by demonstration [208] have relied extensively on Kalman filtering to recursively track objects/features and their locations.

## 15. Visuomotor System

The eye is controlled through six muscles operating in pairs corresponding to the three axes of rotation—superior and inferior rectus pitch the eye up or down; medial and lateral rectus move the eye in yaw; the superior and inferior obliques perpendicular to the other muscles roll the eye. The middle temporal (MT) is responsible for the generation of smooth pursuit eye movements. In smooth pursuit eye movements, tracking continues for a short time after a trajectory change, i.e., the oculomotor system predicts target trajectories. Smooth pursuit is highly accurate in tracking and requires a three-component system: a feedback controller (subject to significant delays), target velocity predictive controller and an inverse oculomotor model [209]. Fast, point-to-point, ballistic eye movements are saccades that intersperse smooth pursuit and are elicited by burst cells which output ballistic motion. Saccades are rapid ≈20 ms and frequent ≈2–3 Hz eye movements generated by an internal visuomotor model. There are several regions of the brain implicated in the generation of saccades—lateral intraparietal area, superior colliculus, basal ganglia, posterior parietal cortex, cerebellum and brainstem regions. The lateral intraparietal cortex predicts the retinal data due to the intended eye movements. The stabilisation of gaze is the core of the oculomotor system in which vision and/or vestibular sensory information is exploited to control eye movements involving cerebellar circuits. The processing of eye position occurs in the superior colliculus at the top of the brainstem which drives saccades [210]. The superior colliculus is itself inhibited by fibres from the substantia nigra pars reticula of the mid-brain. This region is influenced by the cerebral cortex especially the frontal eye field which receives input from the visual cortex. Neural fields of a competitive dynamical neural network may be adopted to determine the next saccade target [209]. A saliency map uses a winner-takes-all strategy to select the next saccade target. Saccades shift the gaze of the eye to scan the visual field sequentially. The primary sensory cortex integrates bottom-up data from the senses and top-down data from higher level systems through attention. The CODE (contour detector) theory of visual attention attempts to integrate space-based and object-based cognitive attention [211]. Attention directs the eyes to information-rich sources to maximise information quality extracted from the environment (salience). It is the visual cortex that selects targets for gazing which de-activates inhibition of the superior colliculus thereby invoking the required saccade. While the eye performs saccades, it is effectively blind — vision processing is performed exclusively during the fixations of around 200 ms in duration. During the saccade, the oculomotor neurons fire maximal frequency bursts which settle to a lower rate during fixation. By monitoring the feedforward command signal to the oculomotor muscles, the oculomotor system computes the eye position. Eye movements are fed back to the brain to enable it to compensate in the construction of internal models of the world. This internal model is constructed from fleeting visual fragments due to the saccades. Despite the impression of a stable world due to this internal model, there is much missing information in its construction which is “filled in” by the brain. 

There are two main head-eye control processes—optokinetic reflex (OKR) and vestibular ocular reflex (VOR). They share cerebellar circuitry of the cerebellar flocculus but sensory data is used differently yet they cooperate to minimise retinal slip error conveyed by climbing fibres in stabilizing gaze—VOR uses vestibular inputs in feedforward mode while OKR uses visual inputs in feedback mode [212]. The OKR involves a negative feedback loop directed by visual data from the eyes mediated by a three-neuron loop involving vision neurons–flocculus interneuron–extraoculomotor neuron. The feedforward VOR uses the vestibular apparatus of the inner ear to detect movement of the head which responds much faster than the optokinetic feedback loop. Retinal imaging is stabilised through compensatory eye movements in response to head movements. The VOR stabilises human gaze to keep the retinal image steady and utilises inertial sensors to measure angular head velocity while vision measures retinal angular position [213]. VOR is a feedforward system mediated by a three-interneuron reflex arc involving vestibular nuclei interneuron–flocculus interneuron–extraocular motoneurons [214]. Purkinje cells of the flocculus receive three kinds of synaptic inputs through parallel fibres: head velocity measured by vestibular sensors, retinal slip measured by the retina and efferent eye velocity motor signals. The retinal slip carried by the climbing fibres to the Purkinje cells is a measure of the error signal used for adapting both OKR and VOR. OKR closes the negative feedback loop using a derivative controller while VOR uses purely feedforward control. The cerebellar flocculus implements a neural network model of the inverse dynamics. The error signal for training the feedforward model is generated by the negative feedback loop. The VOR has a rotational component measured by the three orthogonal semicircular canals and a translation component measured by the otolith organs. The integration of multiple sensory modalities in which each is individually ambiguous yields reduced uncertainty in the combined data. For instance, otolith accelerations measure net linear acceleration $\ddot{x}$ but semicircular canals measure angular velocity w giving an estimate of translational acceleration *a*:

(77) $$a=\ddot{x}-\int w\times g$$

There is evidence that both sensory signals are combined within the rostral fastigial nucleus of the cerebellum and the vestibular nuclei of the brainstem through motion-sensitive neurons to allow the formation of internal models of self-motion [215]. The minimum perceptible angular acceleration of the eyes is ≈0.2°/s^2^ while the minimum linear acceleration detectable by the vestibular system is 6 cm/s^2^. Vestibular reflexes operate open loop and are rapid with a response bandwidth of up to 5 MHz and feedback of vestibular measurements by the semicircular canals imposes a delay of 15–30 ms. Retinal images cannot be fed back to the head movement inputs rapidly so VOR is an open loop feedforward control system. VOR gain (ratio of eye to head velocity) adaptations indicate motor learning in the cerebellar flocculus. By altering the parallel fibre-Purkinje synaptic weights by the climbing fibres, compensatory eye movements can be learned according to retinal slip. The vestibular system does not operate at low velocities so ocular stabilisation is supplemented by the OKR which uses visual information about head movements at 1.0 Hz closed loop feedback rates. OKR stabilises images on the retina through feedback of retinal slip with a delay of 80–100 ms, the duration of an image to cross the retina. OKR has an early direct component for translation and a delayed component for rotation. Optokinetic nystagmus is a characteristic eye movement invoked by an image pattern of continuous motion and contributes to visual stability. Both OKR and smooth pursuit have long latencies of around 100–200 ms. The gaze controller involves feedback based on retinal position error and a feedforward signal based on angular head velocity. The feedforward control system triggers the rapid VOR which in humans has a latency of only 10 ms with head rotation frequencies of 4–6 Hz. The parietal cortex performs coordinate transforms from retinal to spatial coordinates [216]. A general cerebellar decorrelation control algorithm has been proposed which is applicable to VOR [217]. It represents an alternative to the more established feedback error learning system (discussed in the next section). The mossy fibre input comprises a copy of motor commands. VOR may be regarded as a feedforward open loop controller which uses an inverse model controller. The inverse dynamic model is based on stiffness and damping terms of the form $\tau =B\dot{\theta }+K\theta$. In VOR, eye torque output is used as a feedforward torque command without feedback while retinal slip is transformed through a feedback-like gain as a feedback error; in OKR, there is closed loop feedback based on an inverse model without feedforward models (input eye state-eye torque output) [218]. Accurate tracking by the predictor requires an accurate inverse oculomotor model. The vestibular-based signal implements a PD controller while the retinal slip signal implements an integrator, giving a PID controller scheme for the VOR-OKR system. Although VOR is faster, OKR is necessary to eliminate residual errors introduced by VOR and generally compensates when VOR data is incomplete, e.g., when smooth pursuit suppresses VOR. A feedback error learning controller uses the output of the PD feedback pathway as a teacher signal to improve the performance of VOR.

## 16. Feedback Error Learning

**Figure 5:**
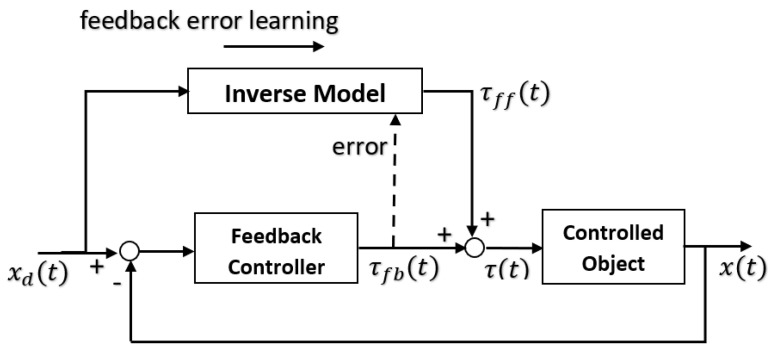
Feedback error learning control system.

Control systems adaptation requires input error signals rather than output error signals that are generated in neural network learning:

(78) $$\frac{dw}{dt}=\eta {\left(\frac{\partial f}{\partial w}\right)}^{T}\tau$$

where *w* = synaptic weights, *f* = nonlinear squashing function, *τ* = total input vector. The learning of models is accomplished through function approximation between sets of input–output data through predetermined local basis functions (radial basis or Gaussian field functions). Motor command error is a hidden variable (not observable) but sensory error is observable but must be converted into motor error to train the inverse model. There are two means for achieving this. Distal supervised learning of inverse models requires the forward predictive model to convert sensory errors into motor commands [219]. Motor command errors are necessary to train the inverse model. Feedback error learning (FEL) uses motor commands from a feedback controller as an error signal by transforming the sensory error into a motor command error which is fed back to train an inverse model (Figure 5). 

FEL feeds the output from the feedback controller as the error to train a neural network model of the inverse dynamics of the system [220]. The sum of feedforward and feedback motor commands act on the physical environment. FEL is based on cerebellar motor control as a form of nonlinear adaptive control with asymptotic hyperstability if $K_{v}^{2}>K_{p}$, *K_p_* > 0 and *K_v_* > 0 [221]. It incorporates an inverse model with an adaptive feedforward component, i.e., it comprises a feedforward and feedback loop. The forward internal model of the system dynamics allows prediction of the motor system state similarly to a Kalman filter. The output of the feedback controller is used as an error signal to train the feedforward controller which models the inverse dynamics. The feedback controller is essentially a self-tuning adaptive controller/sliding controller while the feedforward controller employs a gradient descent to minimise the error. During neural learning, the synaptic weights may be adjusted thus:

(79) $$w\left(t\right)=w\left(t-1\right)+P\left(t\right)x\left(t\right)\left(y\left(t\right)-\hat{y}\left(t\right)\right)$$

where $\hat{y}\left(t\right)=w{\left(t\right)}^{T}x\left(t\right)$ = feedforward predicted eye command output;

$P\left(t\right)=\frac{1}{\lambda }\left(P\left(t-1\right)-\frac{P\left(t-1\right)x\left(t\right)x{\left(t\right)}^{T}P\left(t-1\right)}{\lambda +x{\left(t\right)}^{T}P\left(t-1\right)x\left(t\right)}\right)\;$= covariance of vestibular input *x*;

λ = forgetting factor.

It has been proposed that the cerebellum operates through a FEL mechanism based on feedforward control with feedback from an (inverse) model reference adaptive controller and feedback measurement of muscle position, velocity and acceleration [222]. The forward model computes an efferent copy of the motor command to predict the sensory consequences of those commands. The predicted output from the forward model is passed to a controller to generate the motor commands for the expected effector output. The error is between the predicted and actual sensory output. An evolutionary FEL method based on fuzzy if-then rules was implemented for non-holonomic robotic vehicle control [223]. FEL used the output of a feedback controller as an error to train a neural network forward model. A fuzzy neural model incorporated prior knowledge which used the GA for learning membership function parameters for faster learning than gradient descent. The FEL rule of the form $\hat{u}(t)=u(t-t_{d})+\eta e(t)$ halted training the feedforward controller when the feedback error was zero where *u* = rover control action, *t_d_* = time delay, *η* = learning rate. The fitness function *F*(*t*) for the genetic algorithm was a squared error function: $F(t)=K\left(\gamma +{\left(e(t)\right)}^{2}\right)$ where *K* > 1 and *γ* << 1.

**Figure 6:**
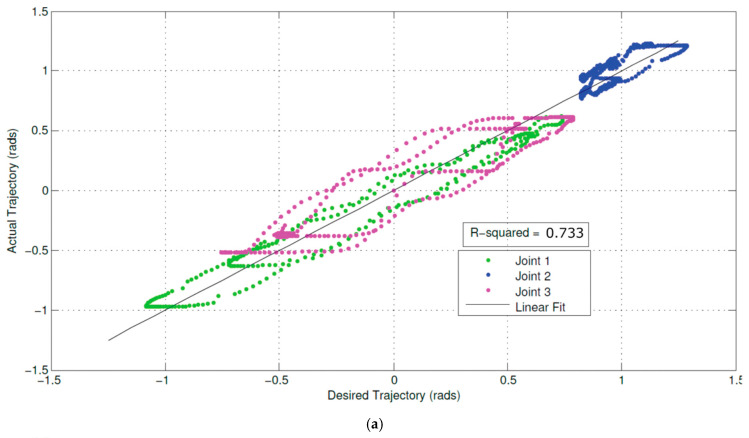

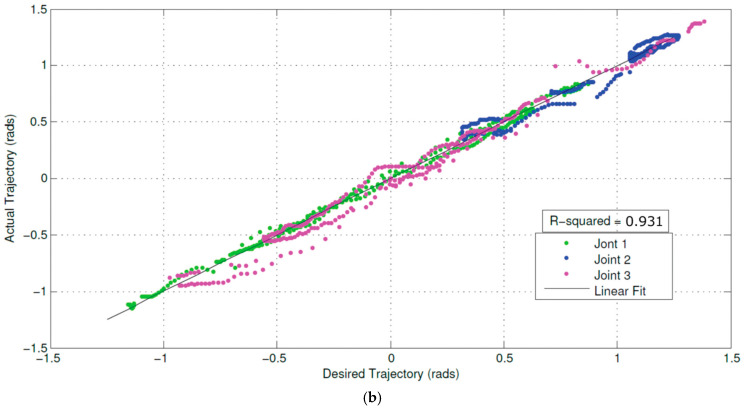
Position error deviation of pan-tilt camera due to (**a**) feedback control alone and (**b**) both feedforward model and feedback control [from Ross and Ellery 2017].

FEL may be implemented through a closed-loop feedback controller computing eye commands based on an inverse model with retinal slip (and slip velocity) and an open-loop feedforward model with vestibular input predicting eye commands (VOR) [224]. A FEL model can track visual targets by slewing a pan-tilt camera mounted onto a manipulator arm simulating planetary rover motion over rugged terrain [225]. It compensated for rover/manipulator motion using a neural network-trained predictor of the pan-tilt dynamics emulating the VOR. The forward model neural network was trained with both a backpropagation algorithm with momentum and an extended Kalman filter algorithm but only the latter could incorporate previous training data. The extended Kalman filter learning rule is given by [226]:

(80) $$w_{i+1}=w_{i}+K_{i}\left(z_{i}-H_{i}w_{i}\right)$$

where $K_{i}=P_{i}H_{i}^{T}{\left(H_{i}P_{i}H_{i}^{T}+R_{i}\right)}^{-1}$= Kalman gain, $P_{i}=F_{i}P_{i-1}F_{i}^{T}+Q_{i}$= covariance, $w_{i}=f\left(w_{i-1}\right)+w_{n}$ = process equation, $z_{i}=H_{i}w_{i}+v_{i}\;$= measurement equation. Under certain restrictive assumptions, this equates to the Ruck learning rate $\eta ={\left(H_{i}P_{i}H_{i}^{T}+R_{i}\right)}^{-1}P_{i}$ [172]. Robot manipulators require control algorithms in which linearised PD controllers tuned by Ziegler–Nichols methods have been dominant. The feedforward model in conjunction with such PD feedback control demonstrated its superiority to PD feedback control alone by reducing pan-tilt mast behaviour dependence on feedback (Figure 6).

This demonstrates the basic principle of forward models on a planetary rover pan-tilt camera mast with and without feedback in slewing the camera to follow visual targets. The adoption of a forward predictive model significantly reduces error excursions. The forward model is used by default and only if significant error occurs is feedback control invoked. This work combines the simplicity of eye movement control (the eye having low inertia) with multi-joint motion of limbs all of which involve the cerebellum. Although the inverse model here is analytic in form, it is learned biologically in the Purkinje cells of the cerebellum [227]. It is learned through cerebellar FEL in which the Cartesian trajectory error is fed back as a joint motor command to train the inverse model representing the transformation from the desired Cartesian trajectory to the joint motor torques [138]. The climbing fibres carry the feedback motor error signals. The inverse dynamic model, once learned, allows motor control to be executed in pure feedforward mode. Forward models are readily updated using supervised learning by comparison of predicted to actual states. Inverse models may be learned by supervised FEL. Yet, there is no evidence of FEL in the cerebellum. However, the OKR is suggestive—it stabilises retinal images based on retinal slip. Climbing fibre inputs to inhibitory Purkinje cells convey feedback motor commands while the Purkinje cells carry sensory error signals in motor command coordinates suggesting that an inverse model of the eye is represented in the ventral parafloccus of the cerebellum [138]. Nevertheless, a recurrent neural network architecture using a forward model yields superior performance to FEL [228]. Notwithstanding this, neural net-based FEL was simulated in controlling a space-based manipulator by updating a feedforward dynamics model [229]. The inverse dynamics of each joint was modelled by its own neural network which used the error from a PD controller to learn its weights. A global feedforward neural network was trained through backpropagation by estimating the feedback error from the controller. This approach reduced tracking errors by 85% compared to PID control due to its ability to compensate for joint flexibility and friction. 

## 17. Manipulator Force Control

Force feedback control will be an essential component in robotic manipulation involving physical contact and interaction with target objects. To incorporate interaction forces of manipulation into forward models represents an order of magnitude greater complexity in forward model suites yet it is crucial to demonstrating its viability for space debris mitigation. The inverse model for manipulation of external forces is given by:

(81) $$\tau =D\left(\theta \right)\ddot{\theta }+C\left(\theta ,\dot{\theta }\right)+G\left(\theta \right)+J^{T}F_{ext}$$

This will require impedance control which exploits the fact that agonist/antagonist pairs of actuators exerting equal but opposing torques will generate no torque but will increase the stiffness of the joint [230]:

(82) $$\tau =J^{T}DK_{f}\left(K_{p}e+K_{v}\dot{e}-F_{ext}\right)-J^{T}D\dot{J}\dot{\theta }+C\left(\theta \dot{,\theta }\right)+G\left(\theta \right)+J^{T}F_{ext}$$

where $e=F^{d}-F_{ext}$, $F=K_{p}e+{K^{\prime }}_{v}{\dot{\theta }}^{d}$. Impedance control in a stiff environment is equivalent to an inner proportional force control loop with an outer PD position controller augmented by feedforward force compensation *J^T^F_ext_*. In this way, impedance control is equivalent to hybrid position/force control [231,232]. Impedance control requires sensory feedback which is subject to time delays—in the case of controlling forces, this can rapidly yield instabilities. Forward models are thus essential to compensate for this:

(83) $$\ddot{\theta }=D{\left(\theta \right)}^{-1}\left[\tau -C\left(\theta ,\dot{\theta }\right)-G\left(\theta \right)-J^{T}F_{ext}\right]$$

FEL adopts the neural net forward model as a reference model in a similar manner to the model reference adaptive control scheme effecting impedance control:

(84) $${\ddot{\theta }}^{d}=K_{f}^{-1}\left(\tau_{ext}+K_{p}\left(\theta_{ref}-\theta \right)+K_{d}\left({\dot{\theta }}_{ref}-\dot{\theta }\right)\right)+{\ddot{\theta }}_{ref}$$

where $\tau_{ext}=J^{T}F_{ext}$. Learning impedance control is also derivable from FEL. During the early stages of learning, impedance control of muscle activity is essential until the internal model has been learned. The impedance controller may operate independently of the forward model to alter the impedance (stiffness) of the limb joints. Joint stiffness defines the gain between the joint torque and deviation from the equilibrium point. Both stiffness and viscosity properties of the human arm vary with applied torque [233]. During early learning phases, when feedback error is large, there is elevated arm stiffness induced by a high gain impedance controller that reduces trajectory errors. This gain decreases until the inverse dynamics has been learned [234]. Muscular joint torque of the human arm is given by:

(85) $$\tau =\tau_{in}+\tau_{ext}=\tau_{in}+J^{T}\left(\theta \right)F_{ext}$$

where $\tau_{in}=D\left(\theta \right)\ddot{\theta }+C\left(\theta ,\dot{\theta }\right)+G\left(\theta \right)$, $J\left(\theta \right)=\frac{\partial q}{\partial \theta }$= Jacobian transform, $\tau_{ext}=J^{T}\left(\theta \right)F_{ext}$= external cartesian force, *D*(*θ*) = inertial term, $C\left(\theta ,\dot{\theta }\right)$ = Coriolis/centrifugal term, *G*(*θ*) = gravitational term. As well as single joint effects, there will be cross-joint effects which are assumed to be negligible. The total muscular joint torque is given by feedforward and feedback components:

(86) $$\tau =\tau_{ff}+\tau_{fb}$$

The feedforward estimate *τ_ff_* represents a learned inverse dynamics model given by:

(87) $$\tau_{ff}=D\left(\theta \right)\ddot{\theta }+B\left(\theta ,\dot{\theta }\right)+G\left(\theta \right)+J{\left(\theta \right)}^{T}F_{ext}$$

The feedback component is given by:

(88) $$\tau_{fb}=K_{p}(\theta^{d}-\theta )+K_{v}({\dot{\theta }}^{d}-\dot{\theta })$$

The joint viscosity gain may be assumed to be related to joint stiffness in such a way as to exhibit high damping at the start and end of the movements for human shoulder and elbow torques *τ*_1_ and *τ*_2_ [235]:

(89) $$K_{v}=\frac{0.42}{\sqrt{{\dot{\theta }}^{T}\dot{\theta }+1}}K_{p}$$

where $K_{p}=K_{\tau }\tau_{ff}+J^{T}\left(\theta \right)K_{ext}J\left(\theta \right)$; 

$K_{\tau }=\left(\begin{array}{cc} 10.8+3.18\left|\tau_{1}\right| & 2.83+2.15\left|\tau_{2}\right|\\ 2.51+2.34\left|\tau_{1}\right| & 8.67+6.18\left|\tau_{2}\right| \end{array}\right)$ Nm/rad;

*K_ext_* = environment stiffness.

Viscosity gives higher damping at the start and end of movements when the velocity is low. Stiffness does not depend on position/velocity rather than being linearly correlated with joint torque *τ*(*θ*). Impedance of a limb exerting an external force on the environment does not depend on whether the force is produced to move the arm or to interact with the environment. The ability to react to the environment rapidly requires the ability to vary the actuator stiffness from full compliance to high stiffness, preferably in conjunction with variable damping. Behaviour-based approaches to robotic manipulation require compliance (low impedance) which is characteristic of the neuromuscular system [236]. Flexibility due to high compliance in joints may be modelled as:

(90) $$\tau_{flex}=\tau_{s}+B_{d}K_{s}^{-1}{\dot{\tau }}_{s}=K_{s}(\theta_{link}-\theta_{motor})+B_{d}({\dot{\theta }}_{link}-{\dot{\theta }}_{motor})$$

where *τ_s_* = spring torque, $K_{s}=\frac{\partial \tau }{\partial t}$ = joint stiffness matrix, *B_d_* = joint damping matrix. Typical joint stiffness for the human shoulder is 45–90 Nm/rad and ankle is 250–400 Nm/rad. In muscle, feedback is delayed suggesting a mechanism is required to compensate for such delays [237]. Stiffness and damping properties independent of feedback may be implemented through feedforward models which compute stiffness in relation to deviation from nominal resting position and zero velocity.

## 18. Preflexive Motor Behaviour

Physical interaction with the world through manipulation or locomotion introduces the requirement for controlling external forces. We initially consider how this is achieved through biological locomotion in animals. Animal behaviour—and the underlying cognition (perception, learning, memory, attention and decision-making) that generates behaviour—has evolved to maximise benefit to the animal in terms of energy efficiency and inclusive fitness [238,239]. Physical morphology and neural control systems have co-evolved together to yield composite behaviours consistent with the physical ecology in which the animal is embedded and this ecology has imposed an evolutionary context for its behaviour. The body and its musculature impose constraints on the movements that can be generated which may be exploited to simplify the control system [240]. Here, we focus on the physical aspects of muscle actuation and its inherent influence on behaviour. A forward model with muscle-like actuation is an example of the coupled interaction between neural computation, the body and the environment [241]. Muscle and tendon acts as a low pass filter by exploiting its stiffness characteristics to eliminate high frequency outputs from neurons which activate the muscle. Muscles may act as wave variable processors, a flexible member to constrain oscillatory waves that are transmitted down the flexible member by imposing a speed limit on transmission [242]. This prevents corrective control action from generating unstable oscillations. Central neural commands sent to the muscles specify position, velocity and force. Feedback signals act as wave variables that add to these central neural commands to form composite descending commands. Unlike neural feedback which imposes time delays, biological muscles and tendons possess inherent physical viscoelastic properties that provide non-neural feedback with immediate (zero order) response [243]. The major role of feedback in muscle control is to augment muscle behaviour by exploiting its inherent stiffness characteristics. This corresponds to using viscoelastic properties of muscle to generates a restoring force for controlled actuation. Preflexes are near zero-order mechanical responses due to passive compliance in elastic structures. Disturbances are rejected within around 20–70 ms overcoming the problem of the time delays of 200–500 ms inherent in proprioceptive feedback control. In fact, mechanical preflexes and sensory feedback are complementary with neural feedback in increasing stability to feedback delays.

The lack of reciprocating mechanisms in biological systems, so characteristic of engineered systems, imposes the need for elastic energy storage. Animal locomotion is characterised by mechanical preflexes in muscles and tendons which serve to store energy elastically as strain energy and release it during the propulsive stroke [244]. Animals exploit the elastic properties of their legs which halves the metabolic energy of running [245], i.e., muscle and tendon elasticity is crucial in energy minimisation of rapid locomotion in reducing the metabolic cost in larger animals. Energy storage as elastic potential energy is stored in the ligaments, tendons and muscles:

(91) $$E=\int F.dx=\int kx.dx=\frac{1}{2}kx^{2}$$

This may be modelled as a linear spring (Hooke’s law):

(92) $$F_{el}=-kx$$

From Young’s modulus of elasticity *Y*:

(93) $$Y=\frac{\sigma }{\varepsilon }$$

where *σ* = *F*/*A* = stress, *ε* = Δ*x*/*x* = strain. Hence,

(94) $$F_{el}=\left(\frac{YA}{x}\right)\Delta x$$

Resonant frequency is given by $w=\sqrt{\frac{k}{m}}$. Elastic energy storage provides a form of non-neural feedback without the delays inherent in sensory neural circuitry. Damping occurs through viscous friction, $F_{\mathrm{fr}}=-b\dot{x}$ where b = viscosity. Mechanical preflexes are enabled by variable muscular viscoelasticity which provides immediate mechanical feedback responses to disturbances from the environment. This viscoelastic preflex reduces instabilities associated with sensory feedback with high gains and time delays. Tendon has a maximum elongation of around 8% but its elasticity is 93% (similar to that of rubber). Damping broadens the resonance curve making it easier to match the resonant frequency to gait frequency of walking. Biological materials such as tendon have curved stress–strain plots giving them high resilience—they have nonlinear elasticity characterised by a J-shaped curve with low resonant frequency for small deflections and high resonant frequency for high deflections which may be modelled as a serial elasticity: $k=\frac{1}{k_{1}}+\frac{1}{k_{2}}+\ldots +\frac{1}{k_{n}}$. The muscles of animal locomotor systems have adjustable stiffness which affects their elastic energy storage capacity [246]. Insects store energy in the protein resilin within the cuticle to drive their wingbeats during flight—the 97% elasticity of resilin provides the basis for high mechanical energy storage and illustrates the utility of compliant structures in robustifying mechanical design. Insect muscles expand/contract only 2% of their lengths with up to 85% of their wing flapping energy being stored elastically. Indeed, asynchronous muscles (which occur in insects only) contract in oscillatory fashion in the insect thorax driving wing actuation. The thorax exhibits high passive stiffness and the delayed stretch activation of muscles gives them higher oscillation frequencies than the activating neural impulses [247]. Feedforward model-based control may be implemented through these musculoskeletal preflexes [248,249]. Indeed, insects may possess forward models to predict the sensory effects of their motor outputs to compensate for the limitations of proprioceptive feedback in rapid flight [250]. Artificial elastomeric materials with high strains may be exploited, e.g., silicone offers properties similar to resilin which is efficient at storing energy for jumping [251]. An insect-like microrobot with elastic hinges rather than rotational motors has been developed which are actuated by electrostatic forces [252,253]. Similarly, Stewart platform hexapods with flexures at each joint have been developed to provide compliant behaviour [254].

## 19. Biological Muscle Modelling

We are concerned with human arm control for which skeletal muscle is the prime mover and possesses viscoelastic properties exploited during manipulation. There are typically multiple sets of muscles recruited to drive the joints of a limb. A performance/cost criterion relates the sum of muscle stresses to the duration of those stresses [255]:

(95) $$C=\sqrt[{p}]{\sum_{j=1}^{m}{\left(\frac{f_{j}}{PCSA_{j}}\right)}^{p}}$$

where *f_j_* = musculo-tendon force of muscle j, $PCSA(mm^{2})=\frac{M(g).\cos\theta }{\rho (g/mm^{3}).L_{f}(mm)}$ = physiological cross-sectional area of muscle, *m* = number of muscles, $\sigma_{j}=\left(\frac{f_{j}}{PCSA_{j}}\right)$ = muscular stress, *p* = power exponent (nominally 2), M = muscle mass, ρ = muscle density = 1.056 g/cm^3^, θ = surface pennation angle (angle between line of action and myofibre long axis) = 0–30°, L_f_ = myofibre length. The m muscles are the source of joint torques which provide n degrees of freedom (where n < m) and exert tensile forces at the endpoints. Skeletal muscle is a linear motor which generates forces by shortening through the parallel sliding of two protein filaments, actin and myosin, through the cycling of cross bridges [256,257]. In animal muscle, force output varies with muscle length according to the overlap of thin and thick muscle filaments [258]. Muscles have spring-like properties that pull joints back to their equilibrium positions by generating a restoring force against perturbations. They have both active and passive components. The passive component is due to nonlinear elasticity and viscous damping; the active contractile component is based on the tension-length relation, force-velocity relation and the neural activation level. Muscle is characterised by its tension-length (stiffness) and force-velocity properties (viscosity) which contribute to muscle torque. Skeletal muscle has a tension-length relation exhibiting increasing force with increasing length and a force-velocity relation exhibiting a rapid force drop with increasing contractile velocity [259]. An inertial muscle component may also be included in its dynamic model:

(96) $$m{\ddot{l}}_{m}=F_{t}cos\theta -cos^{2}\theta \left(F_{act}+F_{pe}+B_{m}{\dot{l}}_{m}\right)+\frac{1}{l_{m}}m{\dot{l}}_{m}^{2}tan^{2}\theta$$

where *F_act_* = maximum active muscle force, *l_m_* = muscle length. However, an inertial component is rarely included due it being negligible. There are many mathematical models that we review here but all attempt to model aspects of its viscoelastic properties. 

The Hill model describes only the force-velocity relation of muscle and not the force-length relation of muscle [260,261]:

(97) c=(F+a)(v+b)

This may be rearranged thus:

(98) $$F=\frac{c}{\left(v+b\right)}-a$$

where *F* = muscle force at velocity *v*, *v* =$-\frac{dl}{dt}$=muscle contraction velocity, 0.12 ≤ *a*/*F* ≤ 0.41 (0.41 for the human tricep), 0.12 ≤ *b*/*v* ≤ 0.41 (0.41 for the human tricep), $a=\frac{P_{\max}}{v_{\max}(1-2\sqrt{P_{\max}/F_{iso}v_{\max}}}$= 1013 ± 684 N = activation level, $b=\frac{P_{\max}}{F_{iso}(1-2\sqrt{P_{\max}/F_{iso}v_{\max}}}$= 0.435 ± 0.181 m/s, $c=\frac{P_{\max}}{(1-2\sqrt{P_{\max}/F_{iso}v_{\max}}}+\frac{P_{\max}^{2}}{F_{iso}v_{\max}{\left(1-2\sqrt{P_{\max}/F_{iso}v_{\max}}\right)}^{2}}$= 2948 ± 1681 W, $P_{\max}=F_{\max}v_{\max}=ab+c-2\sqrt{abc}$, $F_{\max}=\sqrt{\frac{ac}{b}}-a$, $v_{\max}=\sqrt{\frac{bc}{a}}-b$=$\frac{bF}{a}$= maximum muscle shortening velocity that occurs when muscle tension *F* = 0. The maximum shortening velocity varies with muscle type such that it is higher for fast twitch fibres than for slow twitch fibres. There are several variants on the Hill model [262]:

(99) $$F_{v}=\frac{Fkv_{\max}-Fkv}{v+kv_{\max}}$$

where $k=\frac{a}{F}$= shape parameter, $\frac{F_{0}}{F}$≤ 1.3. The output force is dependent on muscle contraction velocity which falls with muscle shortening—it is represented by a steep rectangular hyperbola. Although most muscle models assume linearity, the force-velocity curve is nonlinear with a concave characteristic suggestive of a quadratic relation of the form $F=Bv^{\alpha }$ where *α* = 0.5 [263]. Furthermore, although the stiffness of muscle is nearly linear for small strains despite varying by 50 times in value, the damping variation is nonlinear [264]. The force-velocity curve for skeletal muscle is parabolic with working power given by:

(100) $$P=Fv=F_{max}v_{max}{\left(1-\frac{v}{v_{max}}\right)}^{2}\frac{v}{v_{max}}$$

Minimum *P* = 0 occurs when *v* = 0 and *v* = *v_max_* while maximum *P* occurs when *v*/*v_max_* = 1/3.

The Huxley skeletal muscle model is based on a linear relation between tension and extension due to the existence of cross-bridges between myofilaments which exhibit changes in angle according to tension [265]. Skeletal muscle outputs a force dependent of the length, velocity and degree of activation of the muscle. Tension–muscle length curves exhibit nonlinear behaviour when neurally activated. The Huxley model modifies linear behaviour with a power law relation (of the form $F=Bv^{\alpha }$ where *α* = 2). The isometric force assumption (ab < c) implies zero contraction velocity and activation may be assumed to be at maximum:

(101) $$\left(\frac{F}{F_{0}}\right)=k_{1}{\left(\frac{l}{l_{0}}\right)}^{2}+k_{2}\left(\frac{l}{l_{0}}\right)+k_{3}$$

where *F* = isometric force at length *l*, *F*_0_ = isometric force at resting length *l*_0_ = 193.1 N for human skeletal muscle, 0.65*l*_0_ ≤ *l* ≤ 1.2*l*_0_ where *l*_0_ = 215.9 mm for human muscle, *k*_1_ ≤ −13.43, *k*_2_ ≤ 28.23, *k*_3_ ≤ −13.96 for human skeletal muscle. However, around half the stiffness of muscle is due to actin filaments so muscle stiffness is not proportional to the number of attached myosin heads to actin filaments. The simplest Hill–Huxley muscle model that attributes muscle force/torque to the sum of elastic and viscous effects is given by:

(102) $$F=F_{el}+F_{vis}=k\Delta l+bv$$

where *k* = muscle stiffness, *b* = muscle viscosity = 0.17, Δ*l* = muscle length change, *v* = muscle contraction velocity. Muscle viscosity is around 1% of muscle stiffness. Viscous resistance dissipates the energy of contraction [266]. There are simple Hill–Huxley type models in which muscular contractile fibres exert force depending on the length of the fibre modelled as a stiff spring, the speed of contraction and muscle activation level. Muscle tension applied to the momentum arm of the limb produces the joint torque. The mechanical dynamics of muscle are determined by the ratio of the maximum shortening velocity and elastic stretch, both being proportional to the muscle fibre length. There is proprioceptive feedback from the stretch reflex in the control of muscle stiffness but neural feedback loops exhibit transmission delays limiting its efficacy. The most developed muscle model is the Winters–Stark antagonistic muscle model which includes the torque–velocity, torque–length, neural activity and series–parallel properties [267]:

(103) $$F=F_{\max}\alpha (l_{m}-t^{s})\beta ({\dot{l}}_{m})a(t)$$

where *F*_max_ = peak isometric force, *t_s_* = tendon spring length, *l_m_* = total muscle fibre length, *a*(*t*) = muscle activation level such that 0 ≤ *a*(*t*) ≤ 1, $\alpha =1-{\left(\frac{x-l_{0}}{wl_{0}}\right)}^{2}$= force-length relation, *l*_0_ = optimal muscle fibre length, *w* = operation range parameter, typically 0.65, *β* = force-velocity relation such that: $\left(a_{f}+\beta \right)\left(\frac{v}{v_{m}}+a_{f}\right)=\left(a_{f}+1\right)a_{f}$, *v* = shortening velocity, v_m_ = 2*l*_0_ to 8*l*_0_ depending on fibre type contractile speed, *a_f_* = 0.25. The Winters–Stark model can add a nonlinear asymptotic relation between force and velocity yielding muscle tension given by:

(104) $$\tau (x)=k_{p}x+bv+k_{n}(e^{k_{e}x}-1)$$

where *x* = extension, $k_{n}=\frac{\tau_{\max}}{e^{k}}$, *k* = shape parameter, $k_{e}=\frac{k}{x_{\max}}$, *x*_max_ = displacement at maximum tension *τ*_max_. This models viscoelastic properties with a linear viscoelasticity and a nonlinear exponential stiffness parameter. We consider variations on a simplified Winters–Stark model in more detail later.

A common musculotendon model comprises a hierarchical assembly of a viscoelastic muscular passive element (passive element) in series with an elastic tendon (contractile element) [268]. It may be characterised by a non-isometric viscoelastic muscle model comprising two elastic springs in series, one in parallel with a damping element [269]. The total muscle model comprises an assembly of a Hill–Huxley-type muscle (modelling a force-length-velocity relation) in series with an elastic element (modelling muscle’s active stiffness), all in parallel with another elastic element (modelling muscle’s passive stiffness). The muscle model comprises a passive component due to nonlinear elasticity K_ne_ and muscle viscosity B_m_, and an active contractile component K_a_ which comprises a length-tension relation f(l_m_), velocity-length relation f(v_m_) and normalised activation level α(t). The active component may be modelled through the sliding filament theory where the active force is parabolically related to muscle length over 0.5*l*_0_ ≤ *l_m_* ≤ 1.5*l*_0_ with a maximum at *l_m_* = *l*_0_ where *l*_0_ = tendon slack length. Maximum force is generated when all actomyosin cross-bridges are active when the contraction velocity is zero (isometric tension). In fact, the maximum muscular tension is around 1.8F_o_. Muscle stiffness and viscosity is determined by the degree of activity α(t) (i.e., Ca^2+^ ion concentration) in antagonistic muscle pairs [270]. Muscle activation α(t) is dependent neural input u(t) mediated through Ca diffusion modelled as a first order differential equation:

(105) $$\frac{1}{\tau }u\left(t\right)=\frac{da\left(t\right)}{dt}+\left[\frac{1}{\tau }(\beta +\left(1-\beta \right)u\left(t\right)\right]\alpha \left(t\right)$$

where *u*(*t*) = rectified electromyographic (EMG) activity, $\beta =\frac{\tau_{act}}{\tau_{deact}}$, *τ_act_* = activation time constant, *τ_deact_* = deactivation time constant, $a(t)=\frac{a_{0}+{\left(\rho v\gamma \right)}^{2}}{1+{\left(\rho v\gamma \right)}^{2}}$= muscle activation, *a* and *γ* are muscle properties. 

Muscle activation levels distort the linearity of muscle stiffness-muscle torque is based on its moment arm which depends on the joint angle. A rheological motor model of skeletal muscle with nonlinear viscosity comprised two viscoelastic Maxwell elements and a viscoelastic Voigt element in parallel [271]. Each element had viscoelastic properties of stiffness (length-tension relation) and viscosity (load-velocity relation) but with negligible inertia due to the microscopic size of muscle molecules. Both stiffness and viscosity of muscle possess nonlinear behaviour [272]. A more sophisticated Hill–Huxley-type muscle model exerts a force due to the sum of its passive (*PE*) and active (*CE*) components with viscoelastic components [273]:

(106) $$F_{total}=F_{PE}+F_{CE}$$

where

$$F_{PE}=c{\dot{l}}_{m}+\{\begin{array}{c} 0\;for\;l_{m}<l_{0}\\ k{\left(l_{m}-l_{0}\right)}^{\alpha }\;for\;l_{m}\geq l_{0} \end{array}$$

$$F_{CE}=\{\begin{array}{c} \frac{\alpha (t)F_{l}(l_{m})b(v_{\max}+{\dot{l}}_{m})}{bv_{\max}-{\dot{l}}_{m}}\;for\;l_{m}\leq 0\\ \frac{\alpha (t)F_{l}(l_{m})((f-1)v_{\max}+f(1+b){\dot{l}}_{m})}{(f-1)v_{\max}+(1+b){\dot{l}}_{m}}\;for\;l_{m}\geq l_{0} \end{array}$$

$$F_{l}(l_{m})=\{\begin{array}{c} a_{4}l_{m}^{2}-a_{3}l_{m}^{3}+a_{2}l_{m}^{2}+a_{1}l_{m}+a_{0}\;for\;l_{m}\in (l_{\min},l_{\max})\\ 0\;for\;l_{m}<l_{\min},l_{m}>l_{\max} \end{array}$$

where *l_m_* = muscle length, ${\dot{l}}_{m}$= muscle velocity, *l*_0_ = optimal length where the active force is maximum, *F_l_* = isometric force-length ≈ 100 N, *F_v_* = force-velocity, *α*(*t*) = activation, *v*_max_ = maximum shortening velocity beyond which no force is generated, *b* = steepness of dependence on *l_m_*. Passive stiffness is zero in the contracted state. To ensure a non-zero stiffness at equilibrium, muscles are operated in antagonistic pairs. Typically, F_l_(l_m_)→0 below *l*_min_ = 0.5*l*_0_ and above *l*_max_ = 1.5*l*_0_ rising to a maximum at *l_m_* = *l*_0_. There are muscle models of considerable complexity in which active and passive muscles forces are modelled thus [274]:

(107) $$F_{act}=af_{i}(l)f_{v}(v)F_{\max}$$

where *F*_max_ = isometric force

*a* = neural activation

$$f_{v}(v)=\{\begin{array}{c} 1+7.31v/l+4.06v\;v>0\\ 1+v/l-2.25v\;v>0 \end{array}.$$

*v* = maximum velocity = 0.25 m/s

$$f_{l}(l)=\exp\left(-{\left(\frac{\left|{\left(l/0.05\right)}^{2.83}-1\right|}{0.62}\right)}^{2.37}\right)$$

*l* = optimum fibre length = 0.05 m

(108) $$F_{pass}=f_{p}(l)f_{lv}(lv)f_{t}(l)$$

where

$$f_{p}(l)=\{\begin{array}{c} 0.17(e^{208.2(l-0.036)}-1)\;0.036<l<0.057\\ 13.01+2743(l-0.057)\;l>0.057\\ 0\;l<0.036 \end{array}$$

$$f_{lv}(lv)=\{\begin{array}{c} 0.346ve^{208.2(l-0.036)}\;l<0.057\\ 27.4v\;l>0.057 \end{array}$$

$$f_{t}(l_{t})=\{\begin{array}{c} 2(e^{2000l_{t}}-1)\;l_{t}<0.0013\\ 24.93+53855(l_{t}-0.0013)\;l_{t}>0.0013 \end{array}$$

The exponential form of nonlinearity in muscle does not model extremum behaviours adequately and a sigmoidal form may be more appropriate. 

Tendons are the dominant energy storage over muscle and store energy under load determined by their stiffer elastic modulus [275]:

(109) $$E_{\tan}=\frac{\partial \sigma }{\partial \varepsilon }$$

where σ=Eε= stress according to Hooke’s law (maximum 70 MPa), *ε* = strain, *E* = elasticity tensor such that 5773 < E < 27,614 MPa. The maximum isometric stress within a human muscle is around 40–100 N/cm^2^. Muscle and tendon strains are 6% and 4%, respectively. Tendon force may be given by a simple power law:

(110) $$F=kx^{\alpha }$$

where *k* = stiffness coefficient, *l* = tendon strain, *α* = elastic nonlinearity = 2. Tendon stress is related tendon strain nonlinearly through elastic modulus:

(111) $$\sigma =E\varepsilon^{n}$$

where *n* = 2 and 5800 ≤ E ≤ 27,600. Alternatively, tendon elasticity may be given by a generalised Hooke’s law:

(112) $${\dot{F}}_{t}=K_{t}(F_{l}){\dot{l}}_{t}$$

This variant on Hooke’s law is not linear—it has an exponential spring relation for large strains and a linear relation for small strains.

There are three delayed feedback reflex pathways in muscle—Renshaw feedback, spindle feedback and Golgi tendon organ feedback. Renshaw feedback of motoneuron firing rate, Golgi tendon organ feedback of muscle force and jerk and spindle feedback of muscle stretch (proportional to tension which is proportional to velocity) are dependent on stiffness. Feedback at the spinal level occurs through reflex pathways from Ia sensors from the muscle spindles which are sensitive to stretch [276]. The type 1a sensory nerve fibres transmit signals at a speed of 70–120 m/s, the pulse rate being dependent on the rate of change of muscle fibre length. These are the fastest nerve transmissions of the body, indicating the importance of rapid velocity feedback for damping. Muscle spindles contain passive longitudinal linear spring-like transducers within the muscle in series with the muscle actuator component (intrafusal fibres). The muscle spindle resides in the centre of muscle fibres surrounded by actin and myosin fibres and senses the contraction of the surrounding fibres. They provide sensory stretch feedback within the muscle which modulates the behaviour of the primary muscle locally and directly. This feedback influences reflex gains by altering muscle impedance through its viscoelastic properties [277]. Muscle spindle comprises up to 40% of muscle fibre. A muscle reflex mechanism-based neurocontroller has been implemented which emulates the impedance behaviour of the muscle spindle reflex [278]. The muscle spindle reacts reflexively to displacement changes—this spindle reflex is combined with the motor command which invokes muscle stiffness feedback to generate muscle forces through agonist/antagonist muscles:

(113) $$F=M\ddot{q}+B\dot{q}+K(L+l\Delta q)$$

where *L* =$K_{r}r-(q^{d}-q)$, *K_r_* = reflex gain stiffness coefficient, *r* =$b{\dot{q}}^{1/5}(q-q_{0})$= spindle reflex feedback signals defining length-tension relationship, *b* = spindle damping coefficient. The stretch/unloading reflex is mediated by nonlinear spindle receptors with a time delay of 0.03 s in the reflex loop [279]. Neuromuscular dynamics is also characterised by asymmetric time constants with 10 ms for activation and a time constant of 40 ms for deactivation.

## 20. Manipulator Equilibrium Point Control

The next question concerns how to implement viscoelastic behaviour in a robotic manipulator to generate a given Cartesian trajectory. The equilibrium point hypothesis suggests that limb movements are generated by shifting the limb through a series of equilibrium positions along the desired trajectory. Spinal cord circuitry controls specific patterns of muscle groups in driving the limbs towards the equilibrium points in space [280]. Muscle possesses nonlinear elastic properties whose length-tension relation is modulated by neuromuscular activity as well as their reflexive properties [281]. During contact, the viscoelastic properties are altered by differential coactivation of antagonistic muscle groups of flexors/extensors. Similarly, limb inertia may be altered by changing limb configurations. The Cartesian effector coordinates may be defined as a gradient descent of an objective function which allows the generation of joint trajectories. The objective function is defined as the distance from the current end effector position to the cartesian destination:

(114) $$e\left(\theta ,q\right)=\sqrt{\overset{n}{\underset{i=1}{\sum }}{\left(q_{i}^{d}-f_{i}\left(\theta \right)\right)}^{2}}$$

This gives joint coordinates:

(115) $$d\theta =-s\left(t\right)\frac{\left(q-f\left(\theta \right)\right)J\left(\theta \right)}{\sqrt{\sum^{\;}{\left(q^{d}-f\left(\theta \right)\right)}^{2}}}$$

where *s*(*t*) = scaling factor. Once a series of discrete equilibrium points in joint coordinates have been planned to define the trajectory segments, execution follows by controlling the viscoelastic behaviour of the muscles at the joints. Modulation of contraction in the timing and amplitude of muscle activity regulates the viscoelastic properties of the joints to move between postures without using sensory feedback. This is a statement of the Bernard–Cannon principle of homeostasis whereby the stiffness of agonist/antagonist muscle pairs are maintained at equilibrium operating points.

A Cartesian impedance model of a manipulator describes the external forces *F_ext_* at the end effector in terms of inertial *M*, viscous *B* and stiffness *K* properties of the manipulator driven by muscle joint actuators:

(116) $$M(\theta )\ddot{q}+B\dot{q}+Kq=F_{ext}$$

The spring-like behaviour of muscle defines the limb equilibrium posture according to neuromuscular activity which forms a local minimum of potential. There are also viscous friction effects which are velocity dependent. The equilibrium position is shifted through variation in neuromuscular activity. During human arm trajectories, a series of joint positions are determined by consecutively varying the joint stiffness equilibrium defined by the ratio of tension in the agonist and antagonist muscles of the arm [282]. This gives an asymmetric log-normal velocity shape profile given by:

(117) $$v(t)=A_{1}\Lambda -A_{2}\Lambda$$

where *A*_1_, *A*_2_ = agonist/antagonist activation constants, $\Lambda =\frac{1}{\sigma \sqrt{2\pi (t-t_{0})}}e^{-{\left(\ln(t-t_{0})-\mu \right)}^{2}/2\sigma^{2}}$= neuromuscular impulse response, σ = delay time of log-normal function, *μ* = response time of log-normal function. The transition between equilibrium states is a function of the muscle contraction time and the viscoelastic properties of the arm and muscles. Stiffness and viscosity parameters may be represented as:

(118) $$D(\theta )\ddot{\theta }+C(\theta ,\dot{\theta })+G(\theta )=-B\dot{\theta }-K\Delta \theta +\tau_{ext}$$

The joint stiffness matrix may be converted into Cartesian hand coordinates by:

(119) $$K_{h}={\left(J^{T}\right)}^{-1}\left(K+\frac{\partial J^{T}}{\partial \theta }F_{ext}\right)J^{-1}$$

Stiffness control is implemented through the control of mechanical impedance at the joint by coactivation of antagonistic muscles. Co-contracting muscles are employed to adjust the viscoelasticity of the joints. The agonist-antagonist muscles act as opposing springs that implement the equilibrium position with minimum activation such that equilibrium positions may be varied by varying muscle length and tensile forces [283]. This involves muscular control through parameter *λ* for each muscle pair to generate two opposing torques:

(120) $$\tau_{ext}=sle^{\alpha {\left(\lambda_{ext}-\theta -b\dot{\theta }\right)}^{+}-1)}\;\mathrm{and}\;\tau_{flex}=sle^{\alpha {\left(-\lambda_{flex}+\theta +b\dot{\theta }\right)}^{+}-1)}$$

where *s* = muscle strength, *l* = muscle moment arm, *λ_ex_*_t_ or *λ_flex_* = static threshold of motoneuronal recruitment, *α* = function form parameter, $\bar{\lambda }=\lambda -b\dot{\theta }$ = dynamical threshold of motoneuronal recruitment, *b* = intrinsic muscle damping, *θ* = joint angle. From these opposing torques, the equilibrium position can be determined. Activation of agonistic/antagonistic muscles determines the potential minimum (determined by the *α* ratio) and the potential minimum curvature (determined by the *α* sum). Viscoelasticity may be regarded as a form of peripheral feedback gain in that adjustment of the viscoelastic properties regulate the gain of muscle contraction. This allows control of limb movements through a series of stable equilibrium positions along the end effector trajectory. Equilibrium positions form a kinematic network that expresses joint viscoelasticity in terms of kinematic transformations [284]. The kinematic net comprises a set of Moore–Penrose inverse Jacobians weighted by stiffness/compliance matrices. A set of neural net oscillators tuned to the resonant frequencies of each joint have been applied to the actuation of the robot arms on COG to determine the joint equilibrium points [285].

The elasticity of the muscle is determined by its activation level which determines their length-extension which in turn defines the equilibrium position and stiffness of the joint. This may be modelled as a potential function of the joint angle, the negative derivative of which is the generalised force. Point-to-point movement may be represented as the influence of artificial force fields dependent on the position and velocity of the effector that shift the equilibrium positions along the required trajectory [286]. The potential field defines the trajectory of minimum energy, i.e., through the equilibrium positions. The integral of force represents a potential function which is conservative. Joint force/torque field is the gradient of potential with respect to joint displacement:

(121) F=−gradV(q)

For small displacements, elasticity is assumed linear characterised by a quadratic potential function:

(122) $$V=-\mathrm{Fq}=\frac{1}{2}{\mathrm{Kq}}^{2}$$

As long as K is symmetric, there is no curl to the forces and its behaviour may be described by a potential function with zero curl. Any non-symmetric stiffness component would imply a finite curl $\frac{\partial F}{\partial q}$ due nonlinearities between muscle flexure and extension, in which case a potential function would not constitute an adequate description of such a nonlinear system. Muscular behaviour may be represented as a vector field of forces generated by neural circuits in the spinal cord acting on the joints converging onto equilibrium positions of the end effector [287,288]. Biological motor control systems appear to follow a vector sum of modular muscle output responses which may be represented as a potential field of forces [289,290]. The joint torque may be replaced by a neural trajectory potential field of the form:

(123) $$V(\theta ,\dot{\theta },\ddot{\theta },t)=\sum_{i=1}^{k}c_{i}V_{i}(\theta ,\dot{\theta },\ddot{\theta })=D(\theta )\ddot{\theta }+C(\theta ,\dot{\theta })+E(\theta ,\dot{\theta },\ddot{\theta })$$

where *c_i_* = selection and tuning parameter, *V_i_* = motor primitive field (receptive field)$=-K_{i}(\theta -\theta_{0})e^{-\frac{1}{2}{\left(\theta -\theta_{0}\right)}^{T}K_{i}(\theta -\theta_{0})}$= Gaussian field, *K* = local stiffness, *θ*_0_ = equilibrium position, *E* = environment forces. Force field is a nonlinear function of limb position and velocity:

(124) $$V(\theta ,\dot{\theta },\ddot{\theta })=D(\theta )\ddot{\theta }+C(\theta ,\dot{\theta })+E(\theta ,\dot{\theta },\ddot{\theta })-k_{p}\Delta \theta -k_{v}\Delta \dot{\theta }$$

where *k_p_*, *k_v_* = stiffness/viscosity gains. The force fields generated in the spinal cord are modular and act as building blocks for the control of muscular movement. Each spinal cord control module acts as a vectorial contribution to the force field which is summed to control the tunable spring-like properties of muscle. Motor primitives may be combined to generate complex motor movements [291]. Variation in the vectorial summation of a small number of independent basis force field components can generate a variety of control patterns. The force field primitive may be modelled as dependent on both position and velocity:

(125) $$F_{i}\left(q,\dot{q},t)\right)=s\left(t\right)\phi_{i}\left(q,\dot{q}\right)$$

where *s*(*t*) = scaling factor, $\phi_{i}\left(q,\dot{q}\right)=J^{-T}\left(\theta \right)\tau_{i}\left(\theta \right)$= force field potential. In around 80% of cases, coactivating primitives are summed in their effects, the other 20% implementing a winner-take-all strategy. The overall composite force field is given by:

(126) $$F\left(q,\dot{q},t\right)=\overset{n}{\underset{i=1}{\sum }}s\left(t\right)\phi_{i}\left(q,\dot{q}\right)$$

A Gaussian field potential and its equivalent gradient may be given by:

(127) $$\phi_{i}\left(\theta \right)=-g_{0}e^{\frac{1}{2}{\left(\theta -\theta_{0}\right)}^{T}K_{i}\left(\theta -\theta_{0}\right)}$$

(128) $$\tau_{i}=\nabla \phi_{i}\left(\theta \right)=K_{i}\left(\theta -\theta_{0}\right)\phi_{i}\left(\theta \right)$$

where $K_{i}=\frac{\partial \tau_{i}}{\partial \theta_{i}}$. Hence, a set of joint torques is generated by each spinal module converging to an equilibrium configuration *θ*_0*i*_ with joint stiffness *K_i_*. There are thus two parameters to be defined, *θ*_0_ (position control) and *K_i_* (force control). The superposition of force field primitives may be regarded as a mixture-of-experts network. 

Deviations of Cartesian hand trajectories from equilibrium point trajectories due to low dynamic stiffness at the joints indicate that internal dynamic models are also required [292]. Muscle stiffness is inherently nonlinear in its length–tension curve. The angle–torque curve at a joint shows an accelerating nonlinear relation between angle and torque. The measured stiffness $K=\frac{\partial \tau (\theta )}{\partial \theta }$ at *θ* is higher than the equilibrium stiffness $K_{0}=-\frac{\partial \tau (\theta_{0})}{\partial \theta_{0}}$ at equilibrium position *θ*_0_ predicted from linear assumptions. The distance between measured position and equilibrium position will be under-estimated, i.e., excess stiffness is likely to be imposed. An internal dynamic model is necessary to compensate for this.

## 21. Artificial Muscles through Soft Robotics

Micromachining offers the prospect for combined sensors and actuators based on electromagnetism, electrostatics, piezoelectrics and others [293]. However, we focus here on physical emulation of biological muscles, the province of soft robotics. The mechanical efficiency of muscular contraction is around 45–75% and offers 40–100 W/kg specific power performance, though insect flight muscles offer higher power densities of 60–200 W/kg with a cycle frequency of 30–40 Hz. The mechanical dynamics of muscle are determined by the ratio of the maximum shortening velocity and elastic stretch, both being proportional to the muscle fibre length. On contraction, actomyosin is formed from actin and myosin filaments which shortens by 20–40% on the addition of ATP within 50 ms though higher rates of 1 ms are achievable for insect flight muscles. Nature favours material strength while engineered materials favour stiffness [294]. Biological materials are characterised by highly nonlinear responses to stress—the stress–strain curve of biological material is not linear as it depends on the degree of stretching. J-shaped stress–strain curves characterise materials that are initially soft, but exhibit increases in modulus with applied stress, e.g., tendon stress–strain curve gives a sigmoidal shape—the sigmoidal shape is used in neural network squashing functions. Compliant materials impart strength rather than stiffness and are often exploited to store energy. Soft robotics implements artificial muscles as flexible actuators that integrate actuation, sensing and control by responding mechanically to stimuli [295]. For example, robotic hands may be constructed with fingers with compliant joint hinges to emulate tendons. However, for robust handling, compliance must be adjustable. This requires flexible actuators to act as artificial muscles. There are several types of artificial muscle including shape memory alloys, conducting polymers, dielectric elastomers and ionic polymer metal composites [296]. Most involve sandwiching the polymer between two electrodes. The RoboLobster comprised four pairs of three degree-of-freedom legs, a pair of claws, a tail-like hydrodynamic control surface, antenna-based sensors and neural control system [297]. Nitinol wires exhibiting the shape memory effect have been implemented as artificial muscles for RoboLobster legs [298]. Nitinol shape memory alloy provided the actuation mechanism based on antagonistic pairs of artificial muscles at each leg joint and the body joints activated by electric currents. Antagonistic nitinol wires were excited by PWM signals which allowed graded contractions by varying the proportions of martensite/austenite phases. Electroactive polymers (EAP) have potential as artificial muscles [299,300] and are either ionic or field types depending on whether a flow of ions or an external field controls the movement [301]. They offer high deflection with rapid response [302] but their force output and mechanical energy density are low [303]. They may be used as artificial muscles to implement biomimetic propulsion with polyelectrolyte ionic polymer-metal composites (IPMC) being favoured [304,305]. IPMC represent the most promising technology as artificial muscles for space actuation [306,307]. They are smart composites comprising platinum or conductive polymer electrodes encasing an ionic polymer-metal film soaked in a salt electrolyte which bends in response to an applied electric field. They offer high force output with a high stroke deformation in response to input low voltages ≈10 kV/m for narrow gaps but its cyclicity is limited to a few ms. Conversely, mechanical bending induces an electrical voltage that can be measured. Unfortunately, IPMC require a water-based electrolyte to function—fluids are generally not employed in space as engineering materials. Multilayered silicone dielectric elastomer actuators have been used to power heavier-than-air dual flapping winged micro-aerial vehicles [308]. These artificial muscles with moderate strain in a planar four-bar configuration offered power densities of 600 W/kg with a resonant frequency of 500 Hz. They typically require much higher voltages and power input than piezoelectric actuators. Piezoelectric actuators with low power consumption supplied by solar cells have been employed offering greater thrust-to-weight ratios than biological muscle to drive heavier-than-air micro-quadcopters [309]. However, piezoelectric actuators have no compliance unless coupled with mechanisms such a four-bar linkage. Soft sensing is premised on detecting deformation as tactile feedback but sensor deformation typically occurs over multiple degrees of freedom. Soft materials when deformed, bend, twist, stretch, compress, etc. over multiple (effectively infinite) degrees of freedom rendering inverse kinematics relations challenging to formulate in the case of joints actuated by artificial muscles. Biological joint torque generation differs from robotic joint torque generation as groups of muscles span each joint. The piecewise constant curvature approach to joint flexibility is based on Denavit–Hartenburg transform:

(129) $$T=\left(\begin{array}{cccc} cos\gamma cosks & -sin\gamma & cos\gamma cosks & cos\gamma \left(1-cosks\right)/k\\ sin\gamma cosks & cos\gamma & sin\gamma sinks & sin\gamma \left(1-cosks\right)/k\\ -sinks & 0 & cosks & sinks/k\\ 0 & 0 & 0 & 1 \end{array}\right)$$

where *k* = curvature, *γ* = plane orientation, *s* = arc length. The kinematics (including the Jacobian) and inverse kinematics of a soft actuated manipulator cannot be readily defined because curvatures are not typically piecewise constant. Feedforward neural networks are ideal to map ill-defined mappings between task space and joint space in these cases. Many of the limitations of artificial muscles are illustrated by McKibben artificial muscles. The McKibben artificial muscle is a pneumatic actuator powered by compressed gas within an inner inflatable bladder sheathed within an outer helical braid that contracts lengthwise when expanded radially [310]. It gives high force/weight ratio and the contraction force is determined by the tension-length characteristic given by [311]:

(130) $$F=\frac{P}{4\pi n^{2}}[3{\left(\lambda l_{0}\right)}^{2}-B^{2}]-V_{b}\frac{dW}{dl}$$

where *P* = bladder pressure, *n* = number of helical turns per helical thread, *B* = thread length, $\lambda =\frac{l}{l_{0}}$, *l*_0_ = actuator resting length, *l* = actuator current length, *V_b_* = bladder volume, *dW* = volumetric strain energy density change, *dl* = actuator length. McKibben actuators do not exhibit the force–velocity characteristics of muscle [312]. An artificial nonlinear damping element in series with an elastic element is required for McKibben actuators such as a hydraulic damper. A composite muscle comprising antagonistic contractile McKibben actuator elements for muscle force–length behaviour in series with a hydraulic damper for muscle force–velocity behaviour and an artificial tendon connection to structural elements has been constructed [313]. An artificial model of the Achilles tendon has been developed to emulate tendon’s transmission of forces and for energy storage through F = kl^n^ [274]. Unfortunately, these artificial muscle assemblies are cumbersome. Furthermore, biological viscoelastic properties are limited due to the low stiffness of muscles and tendons requiring internal models to compensate [226]—there are even more severe limitations in artificial muscles. 

There are instances of incorporating physical viscoelastic supplementary mechanisms to traditional electric motors. The series elastic actuator comprises a geared motor serially connected to an elastic spring and a parallel strain gauge force sensor between the motor and the load [314]. A feedback loop applies a specific force using the motor to the load regulated by the high accuracy force sensor. It may be modelled as a second order mass-spring-damper system. The force generated by the motor is given by:

(131) $$F_{m}=F^{d}+\left(F^{d}-F\right)\left(k_{pf}+k_{df}s\right)$$

where *F^d^* = desired force, $k_{pf}=\frac{w_{c}^{2}}{w_{n}^{2}}-1$, $k_{df}=\frac{2\varsigma w_{c}m-b}{k_{s}}$, $\varsigma =\frac{1}{2}\frac{b}{k_{s}}w_{n}$, $w_{n}=\sqrt{\frac{k_{s}}{m}}$, $w_{c}=\sqrt{\frac{k_{s}(k_{p}+1)}{m}}$. It has been used to add active compliance to leg joints during locomotion through local force feedback [315]. The differential elastic actuator is a variant that uses differential coupling between a high impedance sensor and a low impedance spring [316]. The programmable spring actuator was inspired by the series elastic actuator to modulate its stiffness from zero (prior to contact) to high (for stiff environments) levels so essential in both locomotion and manipulation [317]. It comprises a rotary elastic spring actuator/gearbox controlled by measurements from an output shaft angle/angular velocity sensor and the spring acting as a force sensor. A microcontroller-based software PID control loop provides the first control loop based on elastic spring force measurement while a second control loop implements angle-based force control. A variant is to employ two springs, one at each end of the range of motion of the joint allowing the load to move freely close to the midpoint. The springs may be moved along the intervening rail to implement variable stiffness in the joint. It offers integrated PD and force feedback with tailorable spring force profiles programmed into the controller as a lookup table of force-angle entries including zero force, linear, constant force and nonlinear behaviour. Damping profiles may be defined similarly—furthermore, this provides a mechanism for relating velocity and force control. If stiffness *k* is variable, the damping factor must be maintained at critical damping to prevent the injection of excess energy into the system: $b(k)=2\varsigma \sqrt{mk}$ where ς = damping coefficient. Nonlinear viscoelastic force and damping functions may be implemented as force-angle/angular velocity lookup table profiles. Dual profiles may be employed to emulate agonistic–antagonistic muscle pairs. There are several variable stiffness actuators concepts which vary their impedance [318,319]. They typically comprise two actuators (such as antagonist pairs) with a passive elastic component to control the compliance and the equilibrium position of the two actuators. A spring-loaded cam-based lever arm represents an alternative to substitute for one of the actuators [320]. They may be used for safe human–robot interactions at the cost of increased actuator complexity [321]. A two-link manipulator with elastic joints has been constructed from spiral springs in series with conventional electric motors and a connecting tendon to the arm [322]. The electric motor stretched or released the pair of spiral springs attached to the tendon wires fastened to the tip of the link to the arm. Emulation of a Hills–Huxley or Winters–Stark-type muscle model in these devices would lend a biomimetic flavour. Active damping through critically damped PD control prevented oscillation. The series elastic actuator, programmable spring and other hardware “muscles” require measurement feedback and special hardware at the joints. This renders these approaches difficult for a space robot system.

## 22. Electric Motors as Artificial Muscles

Rather than attempting to emulate muscle-tendon directly as artificial muscles, traditional electric motors may be employed that simulate aspects of biological viscoelastic properties. We are no longer constrained by the physical properties of the medium but can tailor the viscoelastic properties through software. The first consideration is then to determine the nature of these potential viscoelastic properties. Contact forces whilst grasping serve to perturb the effector position from the equilibrium position but muscle stiffness can be tuned to restore equilibrium. Such equilibrium-point shifting can compensate for applied loads without inverse dynamics computations [323]. Equilibrium point-based impedance control is position-dependent similar to gain scheduling or high gain adaptive control. Impedance control involves increasing joint stiffness and associated damping through muscle co-activation to impart resistance to perturbations at the equilibrium points. The potential field generated by the viscoelastic properties of the joints imposes regularisation to the multiple muscles at each joint, respectively. Muscular force patterns vary with limb position which map the force field to the limb configuration. We assume that muscle length is determined by joint angles. Muscle redundancy requires a muscle Jacobian to relate muscle extension *l* to joint angle:

(132) $$\dot{l}=J_{m}\dot{\theta }$$

Similarly, joint torque τ is related to muscle tension *F*:

(133) $$\tau =J_{m}^{T}F$$

Muscle coordination generates a smooth sequence of stable positions in space to reach the goal. Muscle force outputs the sum vectorially into a viscoelastic force field. Joint muscle and tendon’s viscoelastic properties pull the joint back to their equilibrium positions by generating a restoring force against perturbations according to length–tension curves—a series of stable equilibrium positions constitutes the desired trajectory. An example of a linear muscle length–tension curve is Hooke’s law of elasticity:

(134) ΔF=KΔl

where *K* = stiffness matrix. In this case, the specific equilibrium position determines the viscoelastic properties of the muscle-tendon system at each joint. For example, the flexspline of the harmonic drive gearing system introduces flexibility into the joint which is nominally assumed to be linear:

(135) $$\tau_{flex}=k\left(\theta_{l}-\theta_{m}\right)$$

where *θ_l_* = angular displacement of load, *θ_m_* = angular displacement of motor. In fact, the flexspline introduces a nonlinear sigmoidal variation of torque with displacement error. Rather than control joint viscoelasticity to generate equilibrium point trajectories, minimisation of deviation from the mid-range of each manipulator joint may be implemented as a potential function:

(136) $$V\left(\theta \right)=k\sum_{i=m+1}^{n}{\left(\theta_{i}-\frac{1}{2}\left(\theta_{i}^{\min}+\theta_{i}^{\max}\right)\right)}^{2}\;\mathrm{and}\;\tau =-\nabla V$$

This resembles the implementation of the Moore–Penrose pseudoinverse for redundant manipulators with joint limits imposed as constraints [324]. DC electric motors typically incorporate integrated Hall sensors for position feedback. PI joint control offers better reference tracking than PD joint control with elastic manipulator joints. We propose a nonlinear model that is linear through most of the joint range except at joint extrema. Traditional DC motor performance may be enhanced by exploiting mechanical resonance in elastic structural components for the rapid storage and release of kinetic energy. DC motors offer high efficiency and high performance in demanding applications such as large stroke, high frequency wing flapping [325]. Joint torque is linearly proportional to muscle stiffness but is also dependent on contraction velocity which may be linear for small velocity changes for isometric muscle behaviour emulated in an electric motor [326]:

(137) $$\tau =J_{eff}\ddot{\theta }+b_{eff}\dot{\theta }+K\theta$$

where *J_eff_* = motor effective inertia, *b_eff_* = motor effective viscosity. Resonance occurs at a frequency *f*_0_ determined by a stiffness *K* given by:

(138) $$K=\frac{\partial \tau }{\partial \theta }={\left(2\pi f_{0}\right)}^{2}J_{eff}\;\mathrm{and}\;b_{eff}=\frac{\partial \tau }{\partial \dot{\theta }}$$

where $b_{eff}\propto \surd K$. The stiffness of the manipulator is dependent on the effective inertia *J_eff_* which in turn determines the power of a DC motor given by:

(139) $$P=\frac{1}{t}\int_{0}^{t}\tau w.dt=\frac{0.5J_{eff}w_{}^{2}}{4t}$$

where *J_eff_* = moment of inertia. Complications will arise due to the incidence of various frictional properties in motors. The simplest friction model is given by:

(140) $$\tau_{f}=\mu \mathrm{sgn}(\dot{q})+b\dot{q}$$

**Figure 7:**
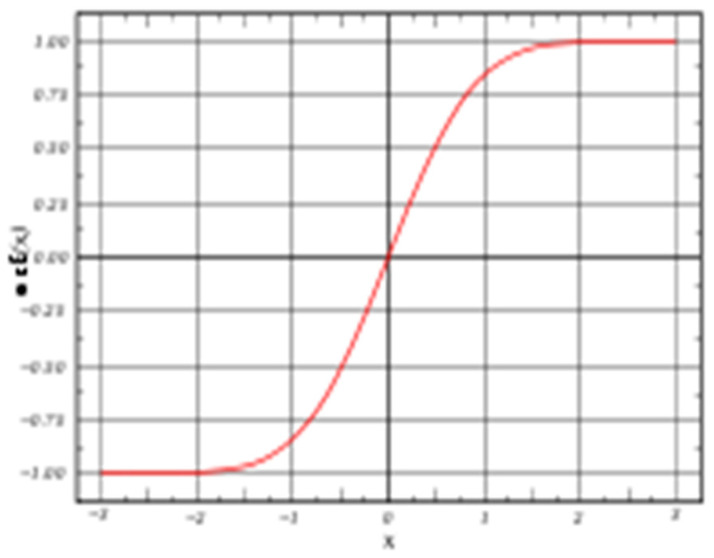
Sigmoidal muscle stiffness behaviour of displacement (vertical axis) against restoring force (horizontal axis).

The first term is Coulomb (static) friction and the second is viscous friction. However, friction is a difficult and complex process to model—even the more complex Stribeck friction model represents a highly simplified model [327]. The viscoelasticity of the human arm is dependent on its specific posture with joint torques dependent on muscle length and contraction velocity [233]. Impedance control utilises the properties of mass-spring-damper to provide stable, low frequency behaviour for adjustable stiffness and damping [234]. The inertial element in muscle is negligible and the viscosity of muscle averages 0.1 Nms/rad in synovial fluid. Our concern here is to provide a tailorable viscoelastic response in the electric motor beyond its physical properties. A rotational variation on the Winters–Stark muscle model is given by [267]:

(141) $$\tau =K\theta +B\dot{\theta }+K_{nlr}(e^{k\theta }-1)$$

where *K_nlr_* =$\frac{\tau_{\max}}{e^{S}}$, *k* =$\frac{\theta_{m}}{S}$, *θ_m_* = displacement at *τ*_max_, *S* = shape parameter indicating concavity, *K* = 120 N/rad for shoulder, *B* = 30 Ns/rad for shoulder. A Gaussian form with a linear slope describes the torque over the joint range:

(142) $$\tau (\theta )=n(e^{-{\left((\theta -\theta_{0})/s\right)}^{2}}+m\theta )\tau_{\max}$$

where *θ*_0_ = joint position at maximum torque, *s* = Gaussian shape function, *m* = slope parameter. A series elastic element gives the concavity of the steep hyperbolic torque-velocity relation which states that the force decreases rapidly at higher velocity—at 1% maximum velocity, tension decreases from maximum force by 5%; at 10% maximum velocity, tension decreases by 35% from maximum force. The rapid drop in force with increasing shortening velocity and rapid rise in force when muscles lengthen which acts as a “brake” to decelerate movement. Linear joint torque viscosity component may be represented as:

(143) $$\tau =\tau \left(\theta \right)-b\dot{\theta }$$

where $b=J^{T}BJ$=joint viscosity. The viscosity parameter is asymmetric to contraction and extension. For contraction, $B=\frac{\left(1+h)\tau (\theta \right)}{\dot{\theta }+h{\dot{\theta }}_{av}}$ while for extension, $B=\frac{\left(1+0.5h)1\tau (\theta )(1.3\tau_{\max}-1\right)}{\dot{\theta }+0.5h{\dot{\theta }}_{av}}$ where ${\dot{\theta }}_{av}={\dot{\theta }}_{\max}-(0.5(1-\frac{\tau (\theta )}{\tau_{\max}})(e^{-{\left((\theta -\theta_{0})/s\right)}^{2}}+m\theta ))$, *h* = Hill shape parameter = 0.1 for slow muscle or 0.5 for fast muscle, ${\dot{\theta }}_{\max}$= unloaded maximum contractile velocity = 2 l_0_/s for slow muscle or 10 l_0_/s for fast muscle. It is plausible that these subtleties could be readily captured in a software model to emulate muscle viscoelastic behaviour in an electric motor. We suggest that power laws do not exhibit linearity for small muscle extensions and that a sigmoidal relation is more appropriate to muscle behaviour—higher neural activity (firing rate) increases the steepness of the sigmoidal form. The sigmoidal form implies that muscle resists lengthening at extrema acting as a brake. We suggest a nonlinear sigmoidal relationship that imposes high restoring forces as the manipulator joints approach their extrema values:

(144) $$\tau =J_{eff}\ddot{\theta }+b_{eff}\dot{\theta }+b\left(\theta_{mid}-\theta \right)\dot{\theta }+K\left(\frac{1}{1+e^{-\left(\theta_{mid}-\theta \right)}}\right)$$

where *θ_mid_* = midpoint of the joint. This gives a viscoelastic restoring force that is absent at or near the midpoint of the joint but grows linearly with excursion away from the midpoint until it becomes extremely high as the extrema are approached (Figure 7): 

Further subtleties of a Winters–Stark model could be captured but may not be necessary for adaptive and robust behaviour in robotic manipulation. The extended or unscented Kalman filter may also be exploited to implement control of both DC and AC electric motors by estimating rotor angular velocity from rotor angle measurements thereby reducing the requirement for multiple joint sensors [328].

## 23. Conclusions

We propose the adoption of two bio-inspired approaches to manipulation to form a three-layered control system for a robotic freeflyer to salvage space debris. The source of bio-inspiration is the human cerebellum and muscle behaviour. The cerebellum provides a forward model which acts as a sensory predictor to generate predictions of the sensory effects of motor actions. Predictive internal feedforward models predict the sensory consequences of motor actions to enable rapid behaviour. Feedforward models define the causal relationship between actions and outcomes. An efference copy of the efferent motor command is input to the predictive forward model which outputs a corollary discharge for comparison with actual sensory inputs. Inverse models define the transformation from sensory signals to motor commands and are required within the feedback controller. Such are used to generate motor commands which are run almost to completion with late proprioceptive feedback used to correct the end movement. Muscle behaviour complements the internal forward modelling by implementing mechanical responses without time delays. 

For space debris manipulation, a set of forward models may be trained for nominal movements with rapid responses—this is essential to control the forces of grappling on acquiring the debris target and then passivating it (we can assume that debris will not be dynamically cooperative). The selected forward model predicts sensory values in response to commands (unlike feedback inverse models that measure sensory values to determine commands). The forward models must be implemented as a set of neural network models to ensure that it is adaptive. Manipulator joint motors may adopt viscoelastic behaviour implemented in software to ensure physically compliant behaviour to tolerate misalignments and errors with zero-order responses. This offers the advantages of artificial muscles without adopting soft actuator materials which are poorly suited to space robotics. Furthermore, software can implement more biologically realistic sigmoidal compliant responses rather than the linear model that would be imposed by physical elasticity. The feedback controller—whether it be hybrid force/position control, impedance control or variations thereof—is invoked only if errors deviate beyond a threshold. This three-layered controller promises to provide sufficient robustness and adaptability to deal with a wide range of target payloads. Robotic manipulation to acquire the target is the first phase of the task—the final phase is disposal. Rather than de-orbiting as a solution, we propose salvaging space debris and converting them into valuable commodities. This is disposal by other means. Salvage recovers high value materials and systems from intact but dysfunctional spacecraft. These involve servicing tasks like bolt handling, flexible object handling (thermal blankets and wiring harnesses) and handling of structural plates and beams for which adaptive/robust manipulation is essential. This enables salvaging of bulk items from the satellite such as solar panels, wiring harness, etc. It is essential to recover everything to prevent debris creation. Once the bulk items have been husbanded, the rest of the unrecovered components of the spacecraft may be smelted into a silumin-like alloy which is powdered or drawn into wires as feedstock for 3D printing facilities (such as selective solar melting) that can print metal products on-demand. This recycles all the spacecraft for re-use while controlling the space debris problem.
